# MEKK2 and MEKK3 orchestrate multiple signals to regulate Hippo pathway

**DOI:** 10.1016/j.jbc.2021.100400

**Published:** 2021-02-09

**Authors:** Jinqiu Lu, Zonghao Hu, Yujie Deng, Qingzhe Wu, Ming Wu, Hai Song

**Affiliations:** 1 The MOE Key Laboratory of Biosystems Homeostasis and Protection, Zhejiang Provincial Key Laboratory for Cancer Molecular Cell Biology and Innovation Center for Cell Signaling Network, Life Sciences Institute, Zhejiang University, Hangzhou, Zhejiang, China; 2 Department of Thoracic Surgery, Second Affiliated Hospital, School of Medicine, Zhejiang University, Hangzhou, Zhejiang, China

**Keywords:** Hippo pathway, LATS, signal transduction, phosphorylation, ubiquitination, tumor necrosis factor (TNF), mitogen-activated protein kinase (MAPK), STRIPAK, YAP, CCM, cerebral cavernous malformations, KO, knockout, MAP3Ks, mitogen-activated protein kinase kinase kinase, MAP4Ks, Mitogen-activated protein kinase kinase kinase kinases, STRIPAK, Striatin-interacting phosphatase and kinase, TNF, tumor necrosis factor

## Abstract

The Hippo pathway is an evolutionarily conserved signaling pathway that controls organ size in animals *via* the regulation of cell proliferation and apoptosis. It consists of a kinase cascade, in which MST1/2 and MAP4Ks phosphorylate and activate LATS1/2, which in turn phosphorylate and inhibit YAP/TAZ activity. A variety of signals can modulate LATS1/2 kinase activity to regulate Hippo pathway. However, the full mechanistic details of kinase-mediated regulation of Hippo pathway signaling remain elusive. Here, we report that TNF activates LATS1/2 and inhibits YAP/TAZ activity through MEKK2/3. Furthermore, MEKK2/3 act in parallel to MST1/2 and MAP4Ks to regulate LATS1/2 and YAP/TAZ in response to various signals, such as serum and actin dynamics. Mechanistically, we show that MEKK2/3 interact with LATS1/2 and YAP/TAZ and phosphorylate them. In addition, Striatin-interacting phosphatase and kinase (STRIPAK) complex associates with MEKK3 *via* CCM2 and CCM3 to inactivate MEKK3 kinase activity. Upstream signals of Hippo pathway trigger the dissociation of MEKK3 from STRIPAK complex to release MEKK3 activity. Our work has uncovered a previous unrecognized regulation of Hippo pathway *via* MEKK2/3 and provides new insights into molecular mechanisms for the interplay between Hippo-YAP and NF-κB signaling and the pathogenesis of cerebral cavernous malformations.

The Hippo signaling pathway plays various roles in the regulation of cellular behavior, organ size, and tissue homeostasis (1). Dysregulation of the Hippo pathway leads to tumorigenesis and diseases (2, 3, 4). The Hippo pathway consists of a kinase cascade, wherein MST1/2 kinases interact with SAV1, and LATS1/2 kinases bind MOB1A/B. Upon stimulation, MST1/2 directly phosphorylate and activate the LATS1/2-MOB1A/B complex, which in turn phosphorylates the transcriptional coactivators YAP and TAZ to promote their cytoplasmic localization and proteasomal degradation (5, 6, 7, 8). When the Hippo pathway is off, YAP/TAZ translocate to the nucleus and interact with transcriptional factors, including the TEAD family, to regulate target genes associated with cell proliferation, apoptosis, and differentiation (9).

The Hippo pathway can respond to a variety of intrinsic or extrinsic signals, such as cell–cell contact, cell adhesion, cell polarity, matrix stiffness, mechanical stress, shear stress, energy stress, osmotic stress, and hormonal signals, such as lysophosphatidic acid (LPA) in the serum, which result in the alteration of LATS1/2 phosphorylation and kinase activity (10, 11, 12, 13, 14, 15, 16, 17). The LATS1/2 activity is central to the Hippo pathway. MST1/2 are not absolutely required for the regulation of LATS1/2. In response to various signals, MAP4Ks (mitogen-activated protein kinase kinase kinase kinases) phosphorylate and activate LATS1/2, which then phosphorylate and inhibit YAP/TAZ (18, 19). TAO kinases have been reported to phosphorylate and activate MST1/2, MAP4Ks, and LATS1/2 (20, 21, 22). Several inhibitory mechanisms for LATS activity have been identified. Src inhibits the Hippo tumor suppressor pathway through tyrosine phosphorylation of LATS1 (23). The protein level of LATS2 is regulated through ubiquitination by the E3 ubiquitin ligase SIAH2, NEDD4, and CRL4DCAF1 (24, 25, 26). NUAK2 positively regulates YAP/TAZ activity by phosphorylating and inhibiting LATS activity to promote tumorigenesis (27, 28).

Previously, we found that inflammatory cytokines, such as TNF or IL-1β, trigger LATS1/2 phosphorylation and YAP/TAZ degradation (29). How LATS1/2 are activated by TNF remains elusive. In this study, we identified MEKK2 (encoded by *MAP3K2*) and MEKK3 (encoded by *MAP3K3*) as the kinases responsible for the TNF-induced LATS activation. MEKK2/3 are serine/threonine kinases belonging to the MAP3K family. MEKK2/3 are capable of activating multiple downstream MAPKs, including ERK1/2, JNK, MAPK/ERK5, and p38 (30). MEKK3 has also been reported to play a critical role in TNF-induced NF-κB activation (31). In addition, the activation of MEKK3-KLF2/4 pathway contributes to CCM (cerebral cavernous malformations) disease (32), which is characterized by abnormal blood vessel formation in the brain due the mutations in any of the three CCM genes (CCM1–3) (33, 34). CCM3 is a subunit of the STRIPAK (striatin (STRN)-interacting phosphatase and kinase) (35) that interacts with GCKIII (germinal center kinase) (36). Recently, STRIPAK protein complex has been demonstrated to integrate upstream signals to control the association and phosphorylation of MST1/2 and MAP4Ks, thus initiating Hippo signaling (37). Here, we showed that MEKK2/3 interact with LATS1/2 and promote their activation in response to various upstream signals. Furthermore, MEKK3 associates with STRIPAK complex through CCM2 and CCM3. Our study suggests that MEKK2/3 mediate TNF and various signals-induced LATS activation to regulate the Hippo pathway.

## Results

### TNF induces LATS kinase activation independent of MST1/2 and MAP4Ks

**Figure 1:**
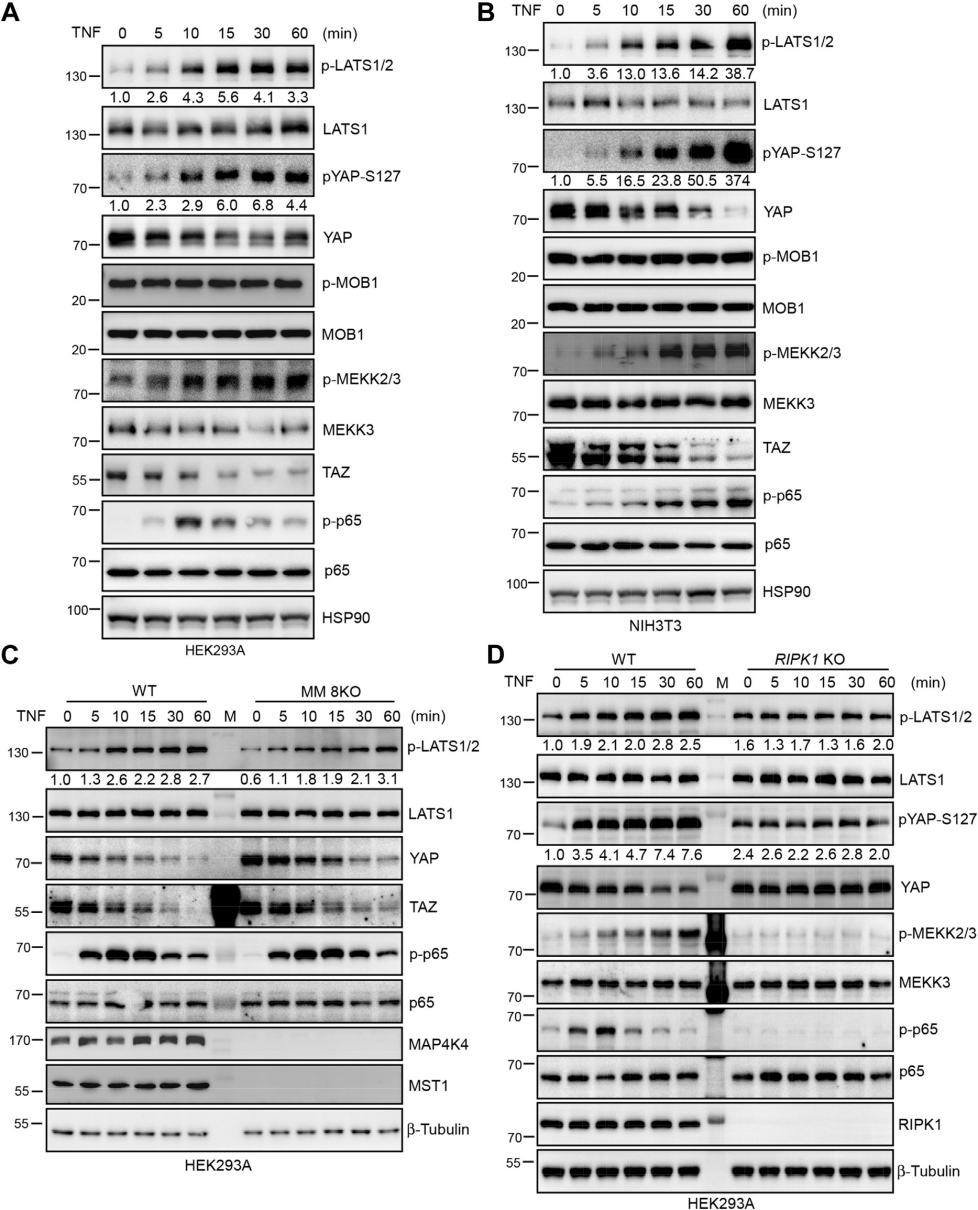
**TNF induces LATS activation independent of MST1/2 and MAP4Ks.***A*, TNF stimulates LATS activation and YAP phosphorylation in HEK293A cells. HEK293A cells were cultured in the presence of TNF for the indicated times and analyzed with indicated antibodies by immunoblotting. *B*, TNF stimulates LATS activation and YAP phosphorylation in NIH3T3 cells. NIH3T3 cells were cultured in the presence of TNF for the indicated times and analyzed with indicated antibodies by immunoblotting. *C*, TNF stimulates LATS activation and YAP phosphorylation in MM 8KO cells. WT and MM 8KO (combined deletion of *MST1/2* and six *MAP4K* genes) HEK293A cells were cultured in the presence of TNF for the indicated times and analyzed with indicated antibodies by immunoblotting. *D*, TNF does not stimulate LATS activation and YAP phosphorylation in *RIPK1* KO HEK293A cells. WT and *RIPK1* KO HEK293A cells were cultured in the presence of TNF for the indicated times and analyzed with indicated antibodies by immunoblotting. The western blot was measured using ImageJ to determine the relative intensities of the p-LATS1/2 and pYAP-S127 bands, which were normalized using the LATS1 and YAP proteins respectively. The relative intensities are shown in (*A–D*).

We previously found that TNF stimulates LATS kinase activation in primary chondrocytes (29). To further investigate the underlying mechanism, we first examined the phosphorylation status of LATS at hydrophobic motif, which is essential for LATS kinase activity using anti-phospho LATS1-T1079/LATS2-T104 antibody in TNF-stimulated HEK293A (Fig. 1*A*), NIH3T3 (Fig. 1*B*), and lung cancer cell A549 (Fig. S1*A*). The phosphorylation of NF-κB subunit p65 validated the successful activation of NF-κB signaling in the TNF-treated HEK293A and NIH3T3 cells (Fig. 1, *A* and *B*). The phosphorylation of LATS1/2 and YAP-S127 (a LATS phosphorylation site) was increased in HEK293A, NIH3T3, and A549 cells (Fig. 1, *A* and *B* and Fig. S1*A*). The phosphorylation of MOB1 Threonine 35 (MOB1-T35) by MST1/2 was unchanged (Fig. 1, *A* and *B*). YAP/TAZ protein levels decreased in conjunction with increased LATS1/2 and YAP phosphorylation (Fig. 1, *A* and *B*), which suggests that TNF elicits LATS activation and subsequent YAP/TAZ inactivation independent of MST1/2. MAP4K family members (MAP4K1/2/3 and MAP4K4/6/7) and MST1/2 have been demonstrated to act direct LATS-activating kinases. The deletion of MAP4Ks and MST1/2 eliminates most of LATS-targeted YAP phosphorylation induced by various signals. Surprisingly, TNF still induced robust LATS phosphorylation and YAP/TAZ degradation in MM 8KO (combined deletion of MST1/2 and MAP4K1/2/3/4/6/7) HEK293A cells (Fig. 1*C*), indicating the existence of additional LATS-activating kinases to mediate TNF-induced LATS activation. To test the dependency of LATS phosphorylation on TNF/NF-κB signaling, we generated *RIPK1* KO HEK293A cells using CRISPR/Cas9 technology. It has been shown that *Ripk1*^−/−^ MEF cells fail to activate NF-κB in response to TNF stimulation (38). Indeed, the phosphorylation of p65 was not activated by TNF treatment in *RIPK1* KO HEK293A cells (Fig. 1*D*). Interestingly, the deletion of *RIPK1* blocked TNF-induced phosphorylation of LATS and YAP (Fig. 1*D*). Previously, we showed that TAK1 is involved in TNF-induced inactivation of YAP independent LATS1/2. TAK1 functions as a pivotal activator to mediate the activation of NF-κB (39, 40). However, whether TAK1 is required for TNF-induced LATS1/2 activation remains unknown. To test this, we generated *Tak1* KO NIH3T3 cells using CRISPR/Cas9. TNF still induced LATS and YAP phosphorylation in *Tak1* KO NIH3T3 cells (Fig. S1*B*). Furthermore, serum starvation and actin depolymerization stimulated LATS and YAP phosphorylation in *Tak1* KO NIH3T3 cells similar as in control cells (Fig. S1, *C* and *D*). These results suggest that TNF stimulates LATS kinase activity and the putative LATS-activating kinases may act downstream RIPK1 and independent of TAK1.

### MEKK2 and MEKK3 associate with LATS1/2 and promote their activation

**Figure 2:**
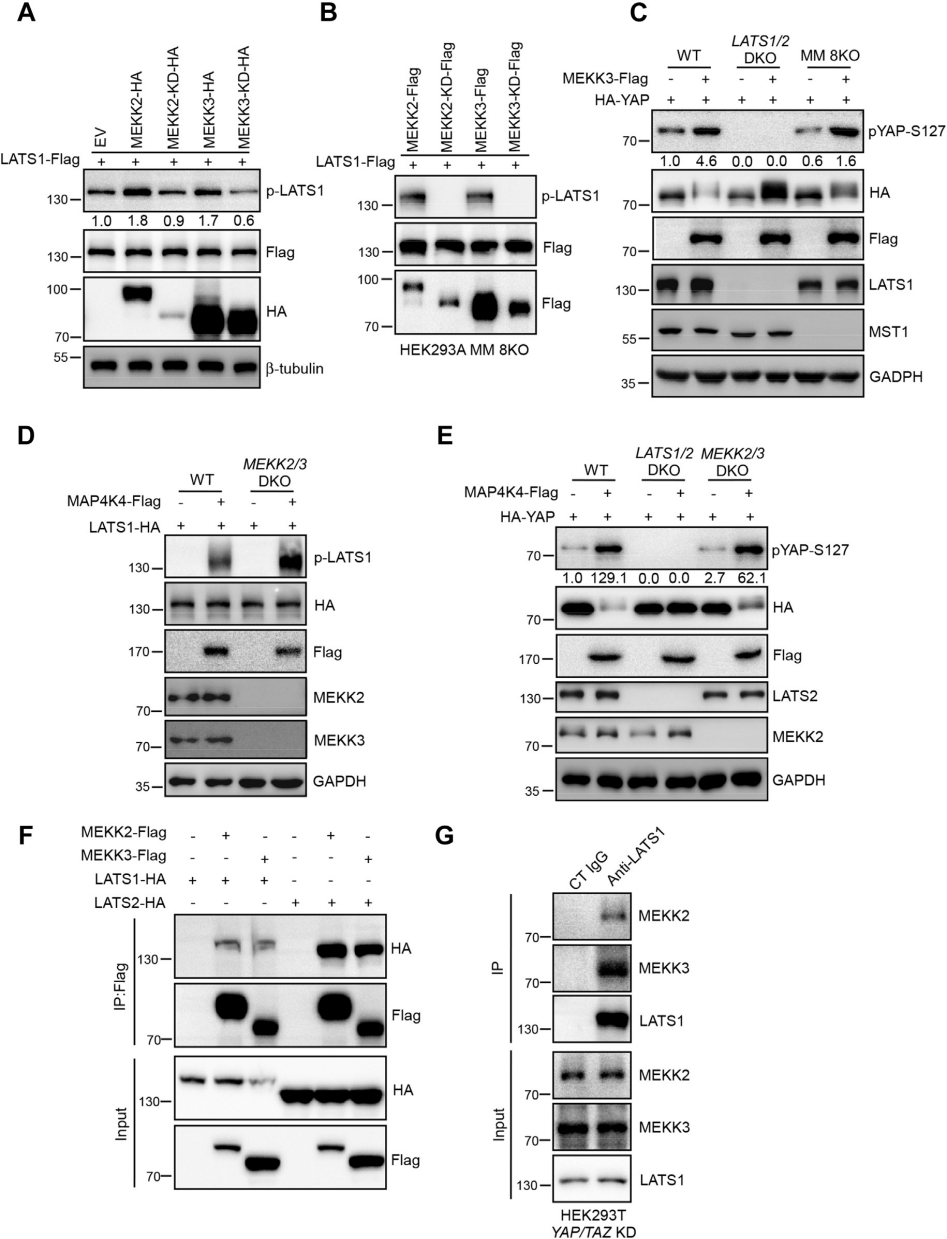
**MEKK2 and MEKK3 associate with LATS1/2 and promote their activation.***A*, MEKK2/3 promote LATS1 activation. HEK293A cells were transiently transfected with LATS1-Myc together with MEKK2-HA, MEKK2-KD-HA, MEKK3-HA, or MEKK3-KD-HA plasmids. LATS1 activation was determined by the phosphorylation status of LATS hydrophobic motif with anti-LATS1-pT1079/LATS2-pT1041 specific antibody. The western blot was measured using ImageJ to determine the relative intensities of the p-LATS1/2 bands, which were normalized using the LATS1-Flag proteins. The relative intensities are shown. *B*, overexpression of MEKK2 and MEKK3 induces LATS1 phosphorylation in MM 8KO HEK293A cells. MM 8KO HEK293A cells were transiently transfected with LATS1-Flag together with MEKK2, MEKK3 or their kinase-dead mutant plasmids. Total cell lysates were analyzed with anti-pLATS antibody. *C*, overexpression of MEKK3 induces YAP phosphorylation in MM 8KO HEK293A cells. WT, *LATS1/2* DKO, and MM 8KO HEK293A cells were transiently transfected with HA-YAP together with vector or MAP4K4 plasmids. Total cell lysates were analyzed with anti-pS127-YAP antibody. The western blot was measured using ImageJ to determine the relative intensities of the pYAP-S127 bands, which were normalized using the HA-YAP proteins. The relative intensities are shown. *D*, MAP4K4 phosphorylates LATS1 in the absence of MEKK2/3. WT and *MEKK2/3* KO HEK293A cells were transiently transfected with LATS1-HA together with vector or MAP4K4 plasmids and analyzed LATS1 phosphorylation by immunoblotting. *E*, overexpression of MAP4K4 induces YAP-S127 phosphorylation in *MEKK2/3* DKO HEK293A cells. WT, *LATS1/2* DKO, and *MEKK2/3* DKO HEK293A cells were transiently transfected with YAP-HA together with vector or MAP4K4 plasmids. Total cell lysates were analyzed with anti-pS127-YAP antibody. The western blot was measured using ImageJ to determine the relative intensities of the pYAP-S127 bands, which were normalized using the HA-YAP proteins. The relative intensities are shown. *F*, MEKK2/3 associate with LATS1/2. HEK293T cells were transfected with MEKK2-Flag, MEKK3-Flag, LATS1-HA, or LATS2-HA. Immunoprecipitated protein complexes by anti-Flag antibody were subjected to immunoblot as indicated. *G*, LATS interacts with MEKK2 and MEKK3 at endogenous level. LATS1 proteins were immunoprecipitated from YAP/TAZ knockdown HEK293T cells. Immunoprecipitants were examined with anti-MEKK2 and MEKK3 antibodies.

To investigate how LATS kinases were activated by TNF, we screened the kinases related with NF-κB pathway to identify candidates that could induce the activation of LATS by coexpressing of candidate kinases with full-length Flag-tagged LATS1 and then examining the phosphorylation status of LATS1-T1079. MST1 was used as a positive control. By this approach, we identified MEKK2 and MEKK3, two highly homologous MAP3Ks (mitogen-activated protein kinase kinase) among 19 MAP3K super family that can efficiently promote LATS1 activation similar as MST1 (Fig. S2*A*). In contrast, other MAP3K family members exhibited no significant kinase activities on LATS1 (Fig. S2, *A* and *B*). Furthermore kinase-dead forms of MEKK2 (MEKK2-KD) and MEKK3 (MEKK3-KD) failed to increase LATS1 phosphorylation (Fig. 2*A*). We next tested whether MEKK2/3 could promote MST1/2 kinase activity and indirectly activate LATS kinase activity. The overexpression of MEKK2 and MEKK3 had no kinase activity on MST1/2 shown by MST1 phosphorylation (Fig. S2*C*) and MOB1-T35 phosphorylation (Fig. S2*D*), a known MST1/2-specific phosphorylation site. Importantly, MEKK2 and MEKK3 still promoted LATS1 phosphorylation in MM 8KO HEK293A cells in a kinase-activity-dependent manner (Fig. 2*B*). Moreover, MEKK3 promoted YAP-S127 phosphorylation in MM 8KO HEK293A cells, but not in *LATS1/2* DKO HEK293A cells (Fig. 2*C*), thereby further confirming that MST1/2 and MAP4Ks are not required for MEKK2/3 to induce LATS and YAP phosphorylation. In addition, we found that MAP4K4 and MST1 efficiently induced LATS and YAP-S127 phosphorylation in *MEKK2/3* DKO HEK293A cells (Fig. 2, *D* and *E* and Fig. S2*E*). Together, these results indicate that MEKK2/3 elicit LATS kinase activity independent of MST1/2 and MAP4Ks.

Since MEKK2/3 promote LATS activation, we tested whether MEKK2/3 are able to interact with LATS kinases. As expected, we observed that MEKK2 and MEKK3 interacted with LATS1/2 at both exogenous and endogenous levels (Fig. 2, *F* and *G* and Fig. S2*F*). Furthermore, the interactions between MEKK2/3 and LATS1/2 were not mediated by YAP and TAZ, since MEKK2/3 still bound to LATS1/2 in *YAP/TAZ* knockdown cells (Fig. S2, *G–I*). In addition, MEKK2 and MEKK3, but not their kinase-dead forms, phosphorylated LATS1 in the *in vitro* kinase assay (Fig. S2*J*). Together, our data suggest that MEKK2/3 act parallel to MST1/2 and MAP4K family kinases to activate LATS1/2 and phosphorylation of YAP in the Hippo pathway.

### MEKK2 and MEKK3 interact with YAP and promote its degradation

**Figure 3:**
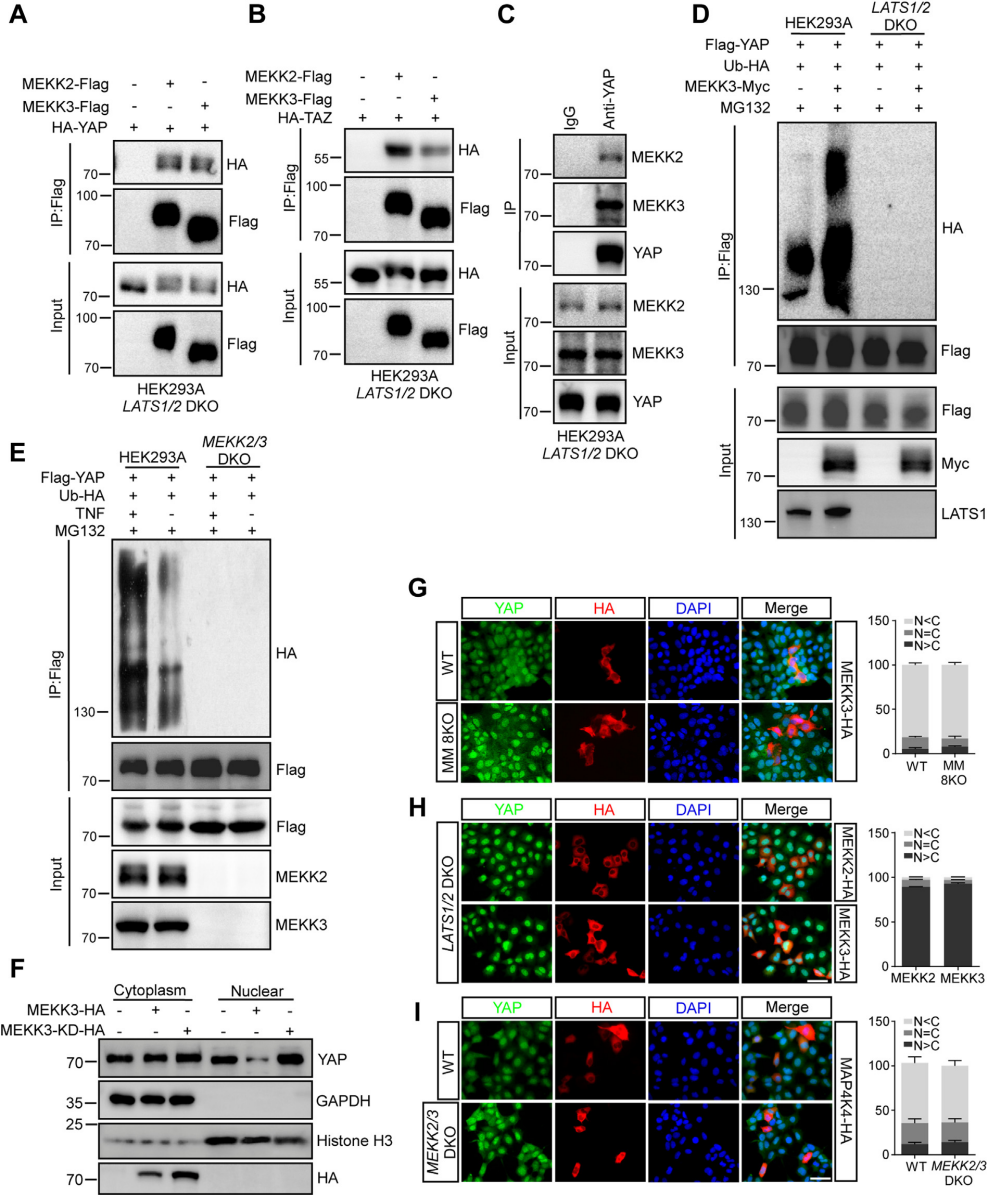
**MEKK2 and MEKK3 interact with YAP and inhibit its function.***A*, YAP interacts with MEKK2 and MEKK3. *LATS1/2* DKO HEK293A cells were transiently transfected with HA-YAP together with vector, MEKK2-Flag or MEKK3-Flag plasmids. MEKK2-Flag or MEKK3-Flag proteins were immunoprecipitated, and the associated HA-YAP proteins were detected by immunoblotting. *B*, TAZ interacts with MEKK2 and MEKK3. *LATS1/2* DKO HEK293A cells were transiently transfected with HA-TAZ together with vector, MEKK2-Flag or MEKK3-Flag plasmids. MEKK2-Flag or MEKK3-Flag proteins were immunoprecipitated, and the associated HA-TAZ proteins were detected by immunoblotting. *C*, YAP interacts with MEKK2 and MEKK3 at the endogenous level. Immunoprecipitants from *LATS1/2* DKO HEK293A cell lysates by anti-YAP antibody were blotted with anti-YAP, MEKK2, or MEKK3 antibodies. *D*, MEKK3-induced YAP ubiquitination is dependent on LATS1/2. Ubiquitination assay of Flag-YAP was performed in WT and *LATS1/2* DKO HEK293A cells with overexpression of HA-tagged Ubiquitin treated with MG132 (10 μM) for 2 h before harvest. *E*, YAP ubiquitination is reduced in *MEKK2/3* DKO HEK293A cells. Ubiquitination assay of Flag-YAP was performed in WT and *MEKK2/3* DKO HEK293A cells with overexpression of HA-tagged Ubiquitin treated with TNF at 5 ng/ml for 2 h and MG132 (10 μM) for 2 h before harvest. *F*, MEKK3 promotes YAP cytoplasmic translocation. HEK293A cells were transduced with Lenti-MEKK3-HA or Lenti-MEKK3-KD-HA virus. Western blot analysis was used to determine the distribution of YAP proteins in the nuclear and cytoplasmic fractions. *G*, overexpression of MEKK3 induces cytoplasmic translocation of YAP in MM 8KO HEK293A cells. WT and MM 8KO HEK293A cells were transiently transfected with MEKK3-HA plasmids. YAP localization was determined by immunofluorescence staining with anti-YAP antibody (*green*). DAPI (*blue*) was used to visualize cell nuclei. Percentage of YAP cellular localization was shown in the *right panel*. Scale bars: 20 μm. A total of 100 HA positive cells were analyzed from three independent experiments in each panel. *H*, MEKK2/3 fail to induce YAP cytoplasmic translocation in *LATS1/2* DKO HEK293A cells. MEKK2-HA and MEKK3-HA were transiently expressed in *LATS1/2* DKO HEK293A. Localization of YAP, MEKK2, and MEKK3 was determined by immunofluorescence staining with the YAP (*green*) and HA (*red*) antibodies. DAPI (*blue*) was used to visualize cell nuclei. Percentage of YAP cellular localization was shown in the *right panel*. Scale bars: 20 μm. A total of 100 HA positive cells were analyzed from three independent experiments in each panel. *I*, overexpression of MAP4K4 induces cytoplasmic translocation of YAP in *MEKK2/3* DKO HEK293A cells. WT and *MEKK2/3* DKO HEK293A cells were transiently transfected with MAP4K4-HA plasmids. YAP localization was determined by immunofluorescence staining with anti-YAP antibody (*green*). DAPI (*blue*) was used to visualize cell nuclei. Percentage of YAP cellular localization was shown in the right panel. Scale bars: 20 μm. A total of 100 HA positive cells were analyzed from three independent experiments in each panel.

It has been reported that human MEKK3 contains a PPXY motif (aa 178–181) providing a binding site for the WW domain of YAP (41). We found that human MEKK2 also contains a PPXY motif (aa 166–169). Since MEKK2/3 also interacted with LATS1/2, we performed co-immunoprecipitation assay to examine the interaction between MEKK2/3 and YAP/TAZ in *LATS1/2* DKO HEK293A cells. YAP and TAZ interacted with MEKK2 and MEKK3 at both exogenous and endogenous levels (Fig. 3, *A*–*C* and Fig. S3*A*). Furthermore, the deletion of WW domain in YAP abolished the interaction between MEKK3 and YAP (Fig. S3*B*). These results indicate that MEKK2/3 interact with YAP/TAZ independent of LATS1/2.

Phosphorylation of YAP/TAZ by LATS1/2 leads to YAP/TAZ cytoplasmic translocation and proteasome-mediated protein degradation. Next, we tested whether MEKK2/3 promote YAP degradation through LATS1/2. MEKK3 expression induced the degradation of endogenous YAP and TAZ in a dose-dependent manner in HEK293T cells, which can be blocked by MG132 administration (Fig. S3*C*). To further explore how MEKK2/3 contribute to YAP degradation, we examined whether MEKK2/3 promote YAP ubiquitination. We found that the expression of MEKK3 markedly enhanced poly-ubiquitination modification of YAP protein (Fig. 3*D*). Of note, MEKK3-induced YAP poly-ubiquitination was abolished in the absence of LATS1/2 (Fig. 3*D*). Conversely, YAP poly-ubiquitination was greatly reduced in MEKK2/3 DKO HEK293A cells (Fig. S3, *D* and *E*). Furthermore, TNF-induced YAP poly-ubiquitination was abrogated in *MEKK2/3* DKO HEK293A cells (Fig. 3*E*). Previous study demonstrates that LATS-induced YAP degradation is through SCF-β-TRCP-mediated ubiquitination (42). Notably, MEKK2/3, but not their kinase-dead mutants, greatly enhanced the interaction of YAP with β-TRCP (Fig. S3*F*). Consistent with the inhibitory function of MEKK2/3 on YAP, the expression of YAP target genes *CTGF* and *CYR61* was decreased in MEKK2 or MEKK3 expressing HEK293A cells, but not their kinase-dead forms (Fig. S3*G*). Furthermore, the expression levels of *CTGF* and *CYR61* were elevated in *MEKK2/3* DKO HEK293A cells (Fig. S3*H*). In addition, we examined the effect of MEKK2/3 on YAP protein localization by immunofluorescence staining. MEKK2/3, but not their kinase-dead mutants, induced endogenous YAP to translocate into cytoplasm in HEK293A cells (Fig. S3*I*), indicating that this process requires MEKK2/3's kinase activity. Nuclear/cytosolic fractionation further supported the notion that MEKK3 promoted YAP cytoplasmic translocation (Fig. 3*F*). Importantly, MEKK3 still elicited YAP cytoplasmic translocation in MM 8KO, but not in *LATS1/2* DKO HEK293A cells (Fig. 3, *G* and *H*), which is consistent with the observation that MEKK3 promoted YAP-S127 phosphorylation through LATS1/2 (Fig. 2*C*). In addition, MAP4K4 stimulated YAP cytoplasmic translocation in *MEKK2/3* DKO HEK293A cells (Fig. 3*I*). Thus, these results indicate that MEKK2/3 promote YAP proteasome-mediated degradation and cytoplasm translocation through LATS1/2.

### MEKK2 and MEKK3 phosphorylate YAP and inhibit YAP-mediated transcriptional activity

**Figure 4:**
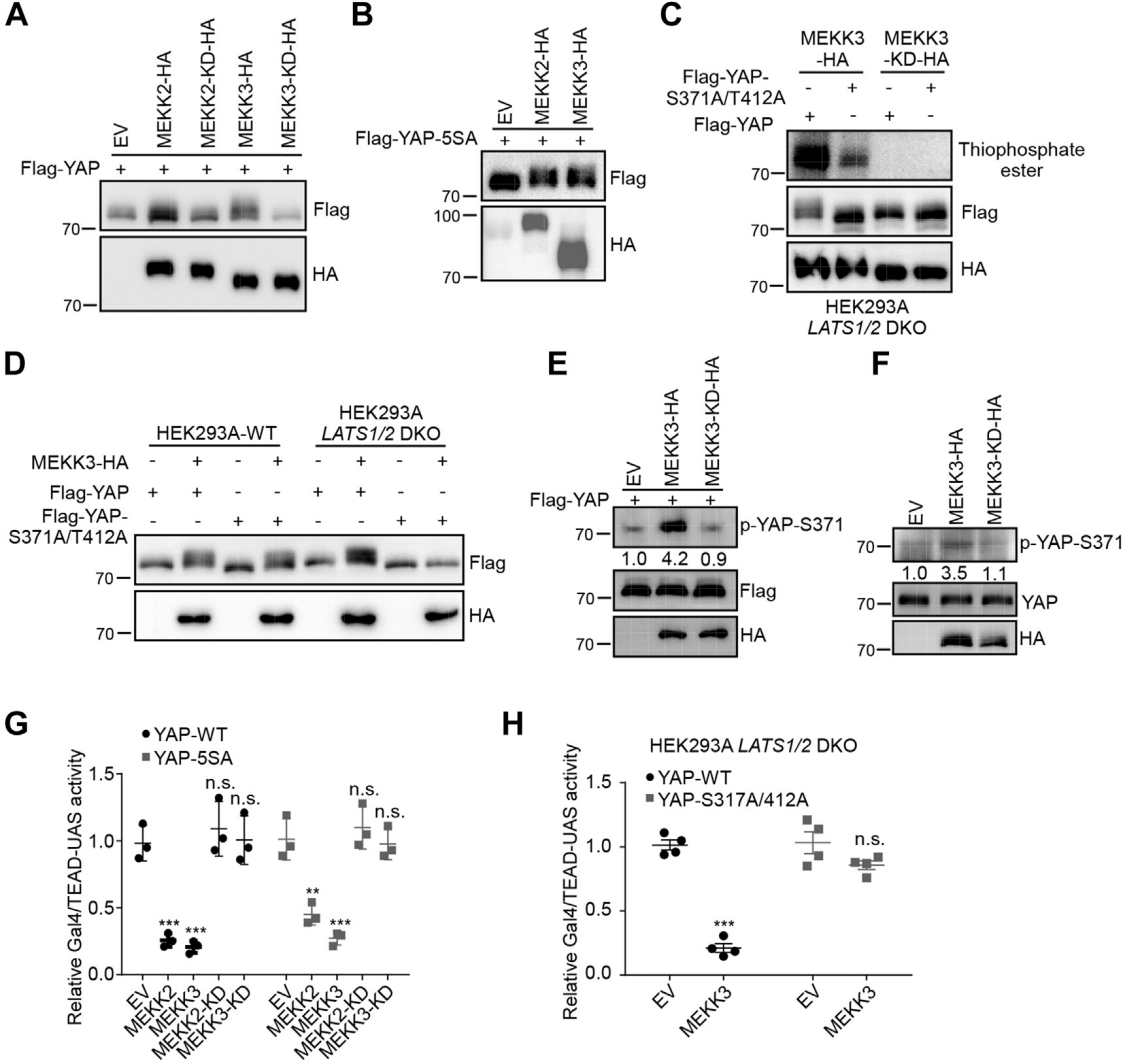
**MEKK2 and MEKK3 phosphorylate YAP and inhibit its transcriptional activity.***A*, MEKK2/3 cause mobility shift of YAP. HEK293A cells were transfected with Flag-YAP together with empty vector, MEKK2-HA, MEKK2-KD-HA, MEKK3-HA, or MEKK3-KD-HA plasmids. YAP mobility shift was shown by immunoblotting. *B*, MEKK2/3 cause mobility shift of YAP5SA. HEK293A cells were transfected with Flag-YAP5SA together with empty vector, MEKK2-HA, or MEKK3-HA plasmids. YAP5SA mobility shift was shown by immunoblotting. *C*, MEKK3 phosphorylates YAP at S371 and T412 *in vitro*. Flag-YAP, Flag-YAP-S371A/T412A mutant, MEKK3-HA or MEKK3-KD-HA was transiently expressed in *LATS1/2* DKO HEK293A cells and immunoprecipitated with anti-Flag or anti-HA antibody individually. An *in vitro* kinase assay was performed using immunoprecipitated Flag-YAP and Flag-YAP-S371A/T412A as substrates in the presence of ATP-γ-S. Total phosphorylation of YAP was detected by immunoblotting with anti-thiophosphate ester antibody. *D*, MEKK3-induced YAP mobility shift is abolished in YAP-S371A/T412A mutant. *LATS1/2* DKO HEK293A cells were transfected with Flag-YAP or Flag-YAPS371-T412A plasmids together with MEKK3-HA. The mobility shift of YAP and YAP-S371A/T412A was shown by immunoblotting. *E*, MEKK3 phosphorylates exogenous YAP at S371. HEK293A cells were transfected with MEKK3-HA or MEKK3-KD-HA plasmids together with Flag-YAP plasmids. Cell lysates were analyzed using anti-YAP S371 antibody. The western blot was measured using ImageJ to determine the relative intensities of the pYAP-S371 bands, which were normalized using the Flag-YAP proteins. The relative intensities are shown. *F*, MEKK3 phosphorylates endogenous YAP at S371. HEK293A cells were transfected with MEKK3-HA or MEKK3-KD-HA plasmids. Cell lysates were analyzed using anti-YAP S371 antibody. The western blot was measured using ImageJ to determine the relative intensities of the pYAP-S371 bands, which were normalized using the endogenous YAP proteins. The relative intensities are shown. *G*, MEKK3 inhibits YAP and YAP5SA-induced Gal4/TEAD4-luciferase activity. Luciferase assay of Gal4/TEAD4 reporter activity was assayed in HEK293T cells transfected with indicated plasmids. N = 3 independent experiments. ∗∗*p* < 0.01, ∗∗∗*p* < 0.001. *H*, MEKK3-mediated inhibition on YAP is compromised in YAP-S371A/T412A mutant. Luciferase assay of Gal4/TEAD4 reporter activity was assayed in *LATS1/2* DKO HEK293A cells. N = 4 independent experiments. ∗∗∗*p* < 0.001.

We always observed a mobility shift of YAP in SDS-PAGE when YAP was coexpressed with MEKK2 or MEKK3 (Fig. 4*A*). The mobility shift of YAP was abolished when treated with λPPase (Lambda Protein Phosphatase) (Fig. S4*A*), indicating that YAP is phosphorylated. MEKK2/3 could activate LATS activity, which may result in the observed phosphorylation of YAP. To rule out this possibility, YAP-5SA, in which all LATS1/2 kinases phosphorylation sites were mutated, was used to coexpress with MEKK2 or MEKK3 in HEK293A cells. However, a remarkable mobility shift of YAP-5SA on SDS-PAGE was still induced in the presence of MEKK2 or MEKK3 (Fig. 4*B*). In addition, MEKK2/3 purified from *LATS1/2* DKO HEK293A cells phosphorylated GST-YAP purified from *E. coli* in the *in vitro* kinase assay (Fig. S4*B*). To identify which amino acids in YAP were phosphorylated by MEKK2/3, we performed mass spectrometric analysis using YAP-Flag proteins purified from *LATS1/2* DKO HEK293A cells coexpressing with either MEKK3-WT or MEKK3-KD. Mass spectrometry showed that phosphorylation of YAP at Ser371 and Thr412 was increased by MEKK3-WT but not its kinase-dead mutant (Fig. S4*C*). Next, we generated YAP mutations targeting Ser371 and Thr412 sites. Mutating the two sites largely eliminated MEKK3-induced YAP phosphorylation in the *in vitro* kinase assay (Fig. 4*C*) and the mobility shift of YAP in *LATS1/2* DKO HEK293A cells on SDS-PAGE (Fig. 4*D*). To further investigate YAP phosphorylation by MEKK2/3, we generated a rabbit polyclonal antibody that specifically recognizes phosphorylated Ser371 of YAP (p-YAP-S371). This antibody recognized GST-YAP recombinant protein incubated with MEKK3-Flag but not MEKK3-KD-Flag immunoprecipitated from HEK293A cells in the *in vitro* kinase assay (Fig. S4*D*), indicating that MEKK3 indeed phosphorylates YAP at Ser371. Furthermore, we were able to detect exogenous and endogenous YAP phosphorylation at S371 by this antibody in MEKK3 overexpressing HEK293T cells (Fig. 4, *E* and *F*). These data indicate that MEKK2/3 directly phosphorylate YAP.

Next, we sought to determine the function of YAP phosphorylation by MEKK2/3. Expression of MEKK2 or MEKK3 significantly inhibited both YAP and YAP5SA-mediated TEAD transcriptional activity in WT HEK293A and *LATS1/2* DKO cells, respectively (Fig. 4*G* and Fig. S4*E*). Notably, the mutations of Ser371 and Thr412 to Ala in YAP largely abolished MEKK3-induced suppression on YAP/TEAD transcriptional activity in a Gal4/TEAD4-luciferase reporter assay (Fig. 4*H*). Furthermore, S371/T412 phosphomimetic form of YAP was phosphorylated at S127 by LATS1 similar as WT YAP (Fig. S4*F*), indicating that the phosphorylation of YAP by MEKK2/3 did not impact its phosphorylation by LATS1/2. In addition, we tested whether the phosphorylation of YAP by MEKK2/3 affects the interaction between YAP and TEAD. However, MEKK2 and MEKK3 did not interfere the interaction between YAP and TEAD in *LATS1/2* DKO HEK293A cells (Fig. S4*G*). Together, our results support the functional importance of MEKK2/3 in inhibition of YAP activity through LATS1/2-dependent and -independent manners.

### MEKK2 and MEKK3 mediate TNF and various signals-induced LATS activation

**Figure 5:**
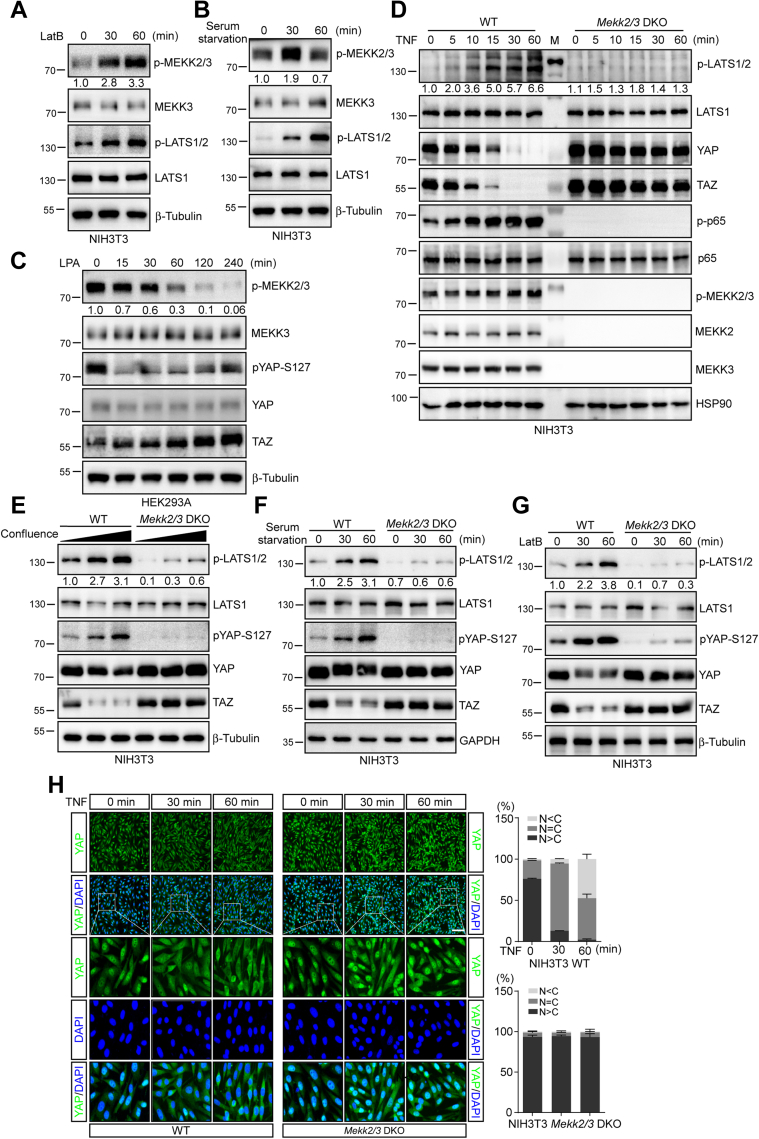
**MEKK2 and MEKK3 mediate TNF and various signals-induced LATS activation**. *A*, actin depolymerization induces MEKK2/3 activation. NIH3T3 cells were treated with 0.2 μg/ml Latrunculin B (LatB) for 30 or 60 min and analyzed with indicated antibodies by immunoblotting. *B*, serum starvation induces MEKK2/3 activation. NIH3T3 cells were depleted with serum for 30 or 60 min and analyzed with indicated antibodies by immunoblotting. *C*, LPA stimulation decreases MEKK2/3 activity. HEK293A cells were serum starved for 6 h, treated with LPA for indicated times, and analyzed with indicated antibodies by immunoblotting. *D*, deletion of MEKK2/3 abolishes TNF-induced LATS activation. WT and *MEKK2/3* DKO NIH3T3 cells were cultured in the presence of TNF for the indicated time and analyzed with indicated antibodies by immunoblotting. *E*, contact inhibition-induced LATS activation and YAP phosphorylation are compromised in the absence of MEKK2/3. WT and *MEKK2/3* DKO NIH3T3 cells were cultured in the presence of LatB for 30 or 60 min and analyzed with indicated antibodies by immunoblotting. *F*, serum starvation-induced LATS activation and YAP phosphorylation are compromised in the absence of MEKK2/3. WT and *MEKK2/3* DKO NIH3T3 cells were depleted with serum for 30 or 60 min and analyzed with indicated antibodies by immunoblotting. *G*, actin depolymerization-induced LATS activation and YAP phosphorylation are compromised in the absence of MEKK2/3. WT and *MEKK2/3* DKO NIH3T3 cells were treated with LatB for 30 or 60 min and analyzed by immunoblotting. The western blot was measured using ImageJ to determine the relative intensities of the pMEKK2/3 and p-LATS1/2 bands, which were normalized using the MEKK3 and LATS1 proteins, respectively. The relative intensities are shown in (*A–G*). *H*, TNF-induced cytoplasmic translocation of YAP is blocked in *MEKK2/3* DKO NIH3T3 cells. WT and *MEKK2/3* DKO NIH3T3 cells were cultured in the presence of TNF for the indicated time. YAP localization was determined by immunofluorescence staining with anti-YAP antibody (*green*). DAPI (*blue*) was used to visualize cell nuclei. Scale bars: 100 μm. A total of 300 cells were analyzed from three independent experiments in each panel.

MEKK3 is an essential kinase to mediate NF-κB signal transduction. The deletion of MEKK3 disrupts TNF-induced NF-κB activation (31). Many stress signals, cytokines, and growth factors induce MEKK2/3 phosphorylation and activation (43). In line with previous observation, TNF stimulation activated MEKK3 kinase activity indicated by the increased Ser526 phosphorylation of MEKK3 (Fig. 1, *A* and *B*). Interestingly, signals such as serum starvation and actin depolymerization, which stimulate Hippo activity, also activated MEKK2/3 kinases as shown by the increased MEKK2/3 phosphorylation (Fig. 5, *A* and *B*). Consistently, LPA, which inhibits Hippo activity, suppressed MEKK2/3 activity (Fig. 5*C*). Next, we tested whether MEKK2/3 are involved in Hippo signaling. As expected, TNF-induced LATS phosphorylation was significantly compromised in *Mekk2/3* DKO NIH3T3 cells (Fig. 5*D*). Intriguingly, cell contact, serum starvation, and actin depolymerization-induced LATS and YAP-S127 phosphorylation was also greatly reduced in *Mekk2/3* DKO NIH3T3 cells and *MEKK2/3* DKO HEK293A cells (Fig. 5, *E*–*G* and Fig. S5, *A* and *B*). The deletion of *Mekk2/3* in NIH3T3 cells resulted in a more profound inhibition on the phosphorylation of LATS and YAP stimulated by various signals. We found that NIH3T3 cells have a higher protein level of MEKK2 and MEKK3, while HEK293A cells express a higher level of MST1/2 and MAP4K4 (Fig. S5*C*). Previously, we have shown that FGF2 induces the phosphorylation of MEKK3 in NIH3T3 cells (44). Therefore, we tested whether FGF2 could activate LATS through MEKK2/3. As expected, FGF2 promoted LATS and YAP phosphorylation in WT NIH3T3, and the deletion of *Mekk2/3* completely abrogated FGF2-induced LATS and YAP phosphorylation (Fig. S5*D*). These results suggest that MEKK2/3 mediate multiple signals to activate LATS1/2.

YAP phosphorylation at Ser127 by LATS1/2 is an important step for regulation of nuclear/cytoplasmic localization, which is one of the most important mechanisms in physiological regulation of YAP activity (10, 45). Previously, we showed that TNF stimulation promotes YAP cytoplasmic translocation (29). Thus, we examined whether TNF-induced YAP protein translocation is regulated by MEKK2/3. YAP was nuclear in almost all NIH3T3 cells and translocated into cytoplasm overtime upon TNF stimulation. However, YAP showed partial and complete nuclear localization in *Mekk2/3* DKO cells even in the presence of TNF (Fig. 5*H*). Together, these results suggest that MEKK2/3 are required for various signals-induced LATS activation and are involved in Hippo-YAP regulation.

### CCM 2 and CCM3 regulate MEKK3 activation through STRIPAK

**Figure 6:**
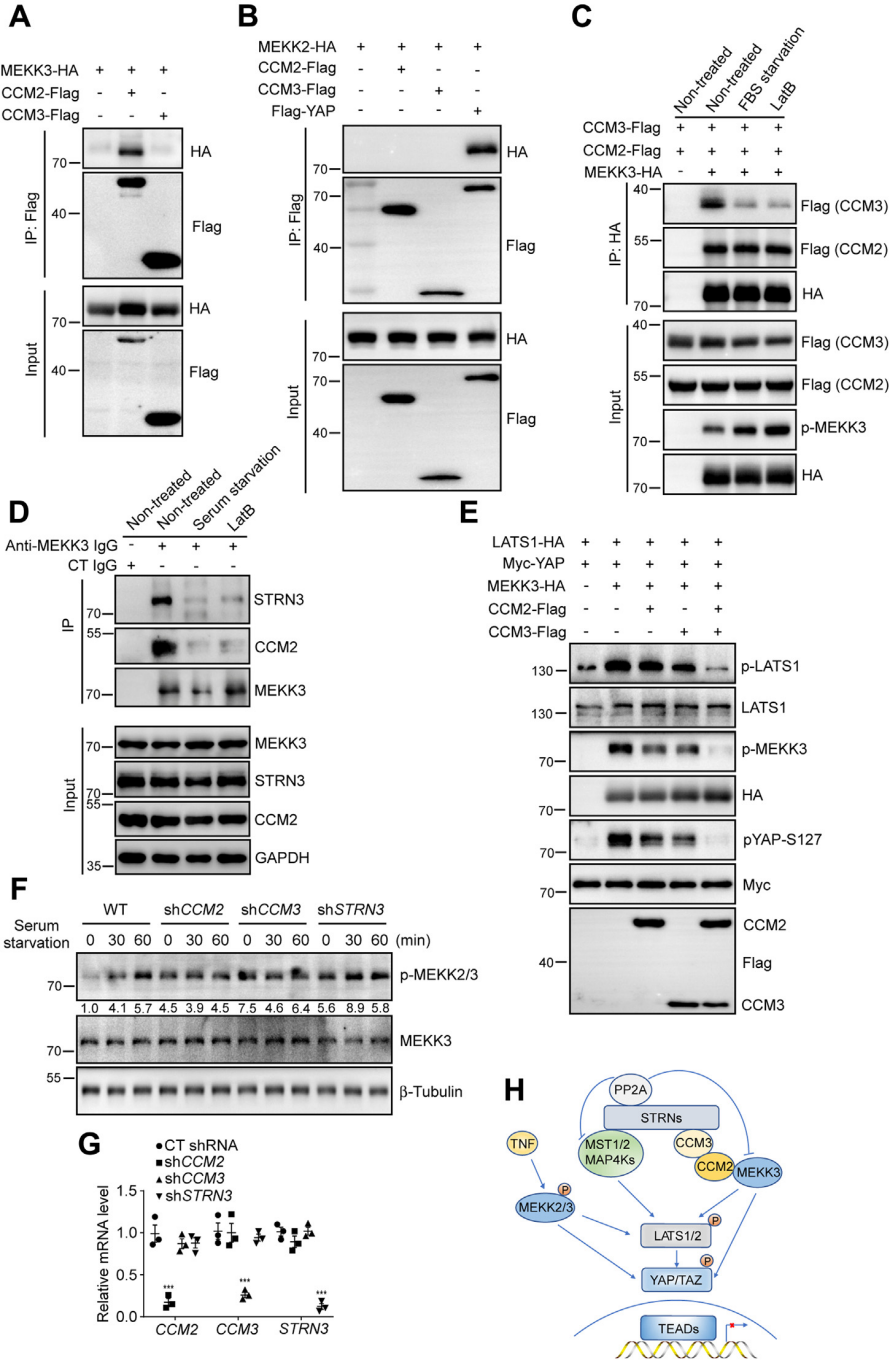
**MEKK3 forms a complex with STRIPAK *via* CCM2 and CMM3.***A*, MEKK3 interacts with CMM2, but not CCM3. MEKK3-HA plasmids were cotransfected with CCM2-Flag or CCM3-Flag into HEK293T cells. CCM2-Flag and CCM3-Flag proteins were immunoprecipitated with anti-Flag antibody, and the associated MEKK3-HA proteins were detected with HA antibody. *B*, MEKK2 does not interact with CMM2 and CCM3. HEK293T cells were transfected with indicated plasmids. CCM2-Flag, CCM3-Flag, and Flag-YAP proteins were immunoprecipitated with anti-Flag antibody, and the associated MEKK2-HA was detected with anti-HA antibody. YAP was used as a positive control. *C*, serum starvation and actin depolymerization disrupt the interaction between MEKK3 and CCM2/3. MEKK3-HA plasmids were cotransfected with CCM2-Flag and CCM3-Flag into HEK293A cells. Cells were serum starved for 2 h or treated with LatB for 1 h. MEKK3-HA proteins were immunoprecipitated with anti-HA antibody, and the associated CCM2-Flag and CCM3-Flag proteins were detected with anti-Flag antibody. *D*, serum starvation and actin depolymerization disrupt the association between MEKK3 and STRN3. HEK293A cells were serum-starved for 2 h or treated with LatB for 1 h. MEKK3 proteins were immunoprecipitated. The immunoprecipitated complex was detected with anti-STRN3 and anti-CCM2 antibody. *E*, CCM2 and CCM3 inhibit MEKK3-induced LATS activation and YAP phosphorylation. HEK293T cells were transfected with indicated plasmids. Total cell lysates were analyzed with indicated antibodies by immunoblotting. *F*, inactivation of CCM2 or CCM3 elicits MEKK3 kinase activity. HEK293A cells were transduced with Lenti-shRNA viruses against *CCM2*, *CCM3*, or *STRN3*. Total cell lysates were analyzed with indicated antibodies by immunoblotting. *G*, quantitative RT-PCR validates the reduced expression of *CCM2*, *CCM3*, and *STRN3* by shRNA-mediated gene knockdown in (*F*). Data are presented as mean ± SD from three technical replicates. ∗∗∗*p* < 0.001. *H*, a proposed model for the regulation of Hippo pathway by TNF and STRIPAK *via* MEKK3. See Discussion for details.

Loss-of-function mutations in CCM family contribute to CCM disease (33, 34). CCM3 is a STRIPAK subunit and interacts with STRIPAK complex core component STRNs and GCKIII (36). Previous study has demonstrated that MEKK3 directly binds to CCM2 (46). Consistent with previous observation, MEKK3 interacted with CCM2, but not CCM3 (Fig. 6*A*). Unlike MEKK3, MEKK2 did not interact with CMM2 and CCM3 (Fig. 6*B*). Recent studies showed that phosphorylated MST1/2 was recruited to STRIPAK complex through SLMAP for inactivation by PP2A (phosphatase 2A) (47, 48). However, we did not observe an interaction between SLMAP and MEKK2/3 (Fig. S6*A*).

Since MEKK3 interacts with CCM2, and CCM2 binds to CCM3, we sought to test whether MEKK3 can form a complex with CCM3 through CCM2 and whether this complex formation is regulated. To test this, MEKK3-HA protein was coexpressed with CCM2-Flag and CCM3-Flag proteins in HEK293A cells. In the immunoprecipitated complex by anti-HA antibody, CCM2-Flag and CCM3-Flag proteins were readily detected by anti-Flag antibody (Fig. S6*B*). Notably, serum starvation or actin depolymerization disrupted the interaction between CCM3 with MEKK3 and CCM2 complex (Fig. 6*C*). Furthermore, we tested whether MEKK3 formed a complex with STRIPAK at endogenous level. MEKK3 proteins were immunoprecipitated from HEK293A cell lysates. Consistently, STRN3 was detected in the immunoprecipitated complex by anti-MEKK3 antibody, and this association was disrupted by serum starvation and actin depolymerization (Fig. 6*D*). Notably, endogenous interaction between CCM2 and MEKK3 was also disrupted during the treatments (Fig. 6*D*). The inconsistency may be caused by the strong interaction between MEKK3 and CCM2 in the overexpression system. MST1/2 and MAP4Ks have been shown to be dephosphorylated and inactivated by PP2A in the STRIPAK complex. To test whether STRIPAK could inactive MEKK3, CCM2 and CCM3 were transfected individually or combinedly with MEKK3. Of note, the phosphorylation of MEKK3 and LATS1 was significantly reduced only when CCM2 and CCM3 were both present with MEKK3 (Fig. 6*E*). In addition, YAP-S127 phosphorylation was abolished in the presence of CCM2/3 and MEKK3 (Fig. 6*E*), which indicates the failed activation of LATS1 kinase activity by MEKK3. Consistently, the removal of CCM2, CCM3, or STRN3 increased the basal phosphorylation of MEKK3, and serum starvation could not further increase the phosphorylation of MEKK3 (Fig. 6, *F* and *G*). Thus, these results indicate that STRIPAK plays an active role in the repression of MEKK3 activity to regulate Hippo pathway through CCM2 and CCM3.

Taken together, our study uncovers that multiple signals, such as TNF and serum starvation or actin depolymerization-induced STRIPAK regulation, converge on MEKK2/3 to modulate LATS1/2 and YAP/TAZ activity (Fig. 6*H*).

## Discussion

The Hippo signaling pathway plays a central role in regulating cell proliferation, cell fate, and tissue size. LATS1/2 are the primary direct kinases phosphorylating YAP/TAZ in the Hippo pathway. Many studies have revealed that the Hippo pathway can integrate various upstream extracellular and intracellular signals to regulate LATS1/2 activation. Our study adds a new dimension to Hippo regulation. Here, we showed that MEKK2/3 interact with LATS1/2 and mediate TNF-induced LATS activation and YAP/TAZ inactivation. Furthermore, MEKK3 associates with STRIPAK complex *via* CCM2 and CCM3. Signals such as serum starvation and actin depolymerization disrupt association between MEKK3 and STRIPAK complex and then activate MEKK3 activity. We propose that STRIPAK plays an active role in the regulation of MEKK3 by relaying upstream signals. Taken together, our work has uncovered a previously unrecognized regulation of Hippo pathway *via* MEKK2 and MEKK3 and provides new insights into molecular mechanisms for the interplay between Hippo-YAP and NF-κB signaling and the pathogenesis of CCM disease.

It should be noted that a large number of LATS-activating kinases were identified in a human kinome screen using an *in vitro* kinase assay in a recent study (18). MEKK2, but not MEKK3, was included in the screening. However, MEKK2 was not identified as a LATS-activating kinase. The initial screen was performed with the truncated form of recombinant GST-LATS1 (aa 638–1130) fusion protein purified from *E. coli* as substrates. In our study, we coexpressed MEKK2/3 with full-length LATS1 in HEK293A cells. MEKK2/3 promoted LATS activation, evidenced by the phosphorylation of LATS in the hydrophobic motif and YAP-S127, a direct LATS phosphorylation site. This process is independent of MST1/2 and MAP4Ks, since MEKK2/3 still promoted LATS and YAP phosphorylation in MST1/2 and MAP4Ks knockout cells. MST1/2 and MAP4Ks phosphorylate the hydrophobic motif of LATS1/2 to activate LATS1/2 (18). MEKK2/3 may utilize different mechanism to regulate LATS1/2 activity. Further studies are needed to elucidate the detailed molecular basis how MEKK2/3-mediated phosphorylation of LATS1/2 ultimately activates LATS kinase activity.

TNF stimulation has been demonstrated to potently activate several MAP4Ks family members, including MAP4K2 and MAP4K3 (49, 50), suggesting that the inflammatory response might activate Hippo pathway *via* MAP4Ks. However, in our study, TNF still induced p65 phosphorylation in MAP4K1,2,3,4,6,7 knockout HEK293A cells similar as in WT control cells. NF-κB pathway is essential for regulating inflammatory and immune responses. In many cases, NF-κB signaling and inflammatory responses activate Hippo signaling. Extensive cross talk occurs between Hippo pathway and innate immunity (51). In *Drosophila*, Hippo (*Drosophila* homology of MST1/2) positively and Yorkie (*Drosophila* homology of YAP/TAZ) negatively regulate the innate defense by regulating the transcription of Cactus (*Drosophila* IκB factor) (52). Similarly, mice that lacked *Yap/Taz* in alveolar type 2 cells exhibited prolonged inflammatory responses in the lung due to the reduced expression of YAP/TEAD target gene IκBa and subsequently increased NF-κB transcriptional activity (53). We have reported a reciprocal antagonism between Hippo-YAP/TAZ and NF-κB signaling during osteoarthritis pathogenesis (29). Inflammatory cytokines, such as TNF or IL-1β, trigger MST1 and LATS1/2 phosphorylation, and TAK1-mediated YAP/TAZ degradation (29). A different mechanism was reported, by which YAP inhibited NF-κB activation through enhancing the degradation of TRAF6 in endothelial cells (54). In addition, MST1 was activated to attenuate TNF-induced inflammatory gene expression after TNF stimulation (55). Here, our study identified MEKK2/3 as mediators for TNF-induced LATS activation, which further provides evidence that the interplay between Hippo-YAP and NF-κB signaling occurs at multiple levels. Additional studies will be necessary to fully appreciate the physiological and pathological functions of MEKK2/3-mediated Hippo regulation.

It has been known that MEKK3 and CCM signaling play critical roles in regulation of the development and vasculature. CCM formation can be genetically rescued by loss of MEKK3 (32). How MEKK3 is activated in CCM mutants remains elusive. Our study brings STRIPAK complex and MEKK3 together to reveal a similar mechanism by which the STRIPAK phosphatase inactivates MST1/2 and MAP4Ks in response to upstream signals. In summary, our study uncovers MEKK2/3 kinases relaying upstream signals, such as inflammation cytokine TNF, serum and actin dynamics, to LATS to regulate Hippo pathway. Further studies are required to elucidate whether CCM/MEKK3-Hippo/YAP pathway is involved in the regulation of CCM formation.

## Experimental procedures

### Cell culture, chemicals, and reagents

HEK293A, HEK293T, NIH3T3, and A549 cells were cultured in Dulbecco's modified Eagle's medium with 10% fetal bovine serum, 100 U/ml penicillin, and 100 μg/ml streptomycin. All the cells were cultured at 37 °C in cell culture incubator with humidified environment in 5% CO_2_. Lipofectamine 3000 (Invitrogen) or polythylenimine (PEI) (Polysciences) transfection reagents were used for plasmid transfection. TNF and FGF2 were purchased from Peprotech. HEK293A (3 × 10^5^/well), and NIH3T3 (1 × 10^5^/well) cells were sparsely seeded on six-well plates and treated with 5 ng/ml TNF or 50 ng/ml FGF2 for different times. MG132 (C2211) was purchased from TargetMol. Lysophosphatidic Acid (LPA) (sc-201053) and Latrunculin B (sc-203318) were purchased from Santa Cruz. Cells were treated with Latrunculin B (200 ng/ml) to disrupt the actin cytoskeleton. We generated a kinase-dead mutant MEKK3-KD, which contains S526/T530 to Ala point mutations, and a kinase-dead mutant MEKK2-KD, which contains S521/T523 to Ala point mutations. The rabbit polyclonal antibody against the phosphorylated of YAP-S371 was produced using the synthetic phosphorylated peptides PGM-S(p)-QELRTMT-C as antigen and purified on a phosphopeptide column.

### CRISPR/Cas9-mediated genomic editing

Guide RNA sequences targeting RIPK1, TAK1, MEKK2, and MEKK3 were cloned into plasmid PX459 (Addgene #62988). Constructs were transfected into HEK293A or NIH3T3 cells by PEI transfection reagent. Twenty-four hours after transfection, cells were selected by puromycin (1.5 μg/ml) for 72 h. Single colonies were picked and characterized by immunoblotting. Guide RNA, shRNA, and siRNA sequences are listed in Table S1.

### RNA extraction and quantitative RT-PCR

Total RNA from cultured cells was isolated using TRIzol Reagent (Invitrogen) according to the manufacture's protocol. Two microgram total RNA was reversely transcribed using SuperScript III Reverse Transcript Kit (Invitrogen). Quantitative PCR was then performed using SYBR Green 2X PCR Master Mix (Yeasen Biotech) on an Applied Biosystems 7900 system (Applied Biosystems). Target gene threshold cycles (Ct values) were normalized to *GAPDH*. The sequences of the primers are listed in Table S1.

### Luciferase assay

HEK293T cells were transfected with pGL3-basic, Gal4/TEAD4-luciferase reporter plasmid together with pRL-TK vector (Promega) as reference control using PEI (Invitrogen). Cells were subjected to luciferase activity measurement as described in dual luciferase reporter assay kit (Promega).

### SDS-PAGE and immunoblot analysis

Equal amount of proteins was loaded into each well and separated by SDS-PAGE. After blocking in 5% milk or 3% BSA, the membrane was incubated with primary antibody at 4 °C overnight. The membrane was washed and subjected to incubation with the HRP-conjugated secondary antibody for 1 h at room temperature. Western blotting images were captured by ChemiScope5300 (Clinx) with ECL substrate. Antibody information is described in Table S2.

### Immunoprecipitation

After transfection with indicated plasmids for 24–36 h, cells were lyzed in a lysis buffer containing 50 mM Tris (pH 7.4), 150 mM NaCl, 1% Triton NP-40, and 1 mM EDTA with protease and phosphatase inhibitors on ice. Twenty microliter anti-Flag M2 magnetic beads or anti-HA magnetic beads were added to the lysates and incubated at 4 °C overnight under gentle agitation. The beads were washed with washing buffer for five times. Finally, the beads were eluted with 2× SDS loading buffer. The eluted proteins were analyzed by SDS-PAGE and followed by immunoblot analysis.

### Cytosolic/nuclear fractionation

Cells were lysed in 100 μl Buffer A (10 mM HEPES pH 7.5, 10 mM KCl, 1 mM EDTA, 1 mM DTT) with protease inhibitors cocktail at 4 °C for 15 min. Lysates were cleared by centrifuge at 10,000*g* for 3 min at 4 °C. The supernatant was cytoplasmic fraction. Nuclear pellets were washed in Buffer A, 5 min for three times, and resuspended in 100 μl Buffer B (20 mM HEPES pH 7.9, 400 mM NaCl, 1 mM EDTA, 1 mM DTT, 10% glycerol, 1%Triton X-100) at 4 °C for 10 min. The supernatant was nuclear fraction after centrifuging at 10,000*g* for 5 min.

### Ubiquitination assay

HEK293T cells were transfected with plasmids expressing Flag-YAP, MEKK3-Myc or HA-ubiquitin. Cells were pretreated with MG132 at 10 μM for 2 h and then treated with TNF at 5 ng/ml for 1 h. Cell lysates were harvested with RIPA buffer (50 mM Tris pH 7.4, 150 mM NaCl, 1%TritonX-100, 1% sodium deoxycholate, 0.1% SDS, protease and phosphatase inhibitors) and incubated with 20 μl anti-Flag M2 magnetic beads at 4 °C overnight. The beads were washed for five times. Bound proteins were boiled in 2× SDS loading buffer and analyzed by SDS-PAGE followed by immunoblot analysis.

### *In vitro* kinase assay

HEK293T cells were transfected with Flag-tagged MEKK3 plasmids and then lysed in RIPA buffer. Flag-tagged MEKK3 proteins were purified by anti-Flag antibody-conjugated magnetic beads. GST-tagged YAP proteins were purified from *E. coli* by glutathione agarose slurry. Purified proteins were washed with kinase washing buffer (40 mM Hepes and 200 mM NaCl, pH 7.5) three times and once with kinase assay buffer (30 mM Hepes, 50 mM KAC and 5 mM MgCl_2_, pH 7.5). Purified kinase and YAP proteins were mixed with ATP-γ-S (500 μM) or ATP (500 μM) in kinase assay buffer. After 1 h kinase reaction at 30 °C, EDTA (final concentration 20 mM, pH 8.0) was added to terminate the reaction at 30 °C for 5 min. PNBM (Abcam, ab138910) (2.5 mM) was added at 25 °C for 40 min to form a thiophosphate ester side chain in the reaction with ATP-γ-S. Western blot was performed using anti-YAP-S371 phospho-specific antibody or anti-thiophosphate ester antibody to analyze the kinase activity.

### Immunofluorescence and imaging analysis

HEK293A or NIH3T3 cells were plated on coverslips. Cells were transfected with plasmids by PEI or treated with TNF at 5 ng/ml for 0, 30, and 60 min and were then fixed with 4% PFA on ice for 20 min. After fixation, cells were then washed with PBST (PBS, 0.1% Triton X-100), followed by blocking with 3% BSA at room temperature for 30 min. Cells were incubated with primary antibodies diluted in 3% BSA overnight at 4 °C. After three washes with PBST, cells were incubated with secondary antibodies in 3% BSA for 1 h. Nuclei were stained with 0.5 μg/ml of DAPI at room temperature for 10 min. Cells were mounted with ProLong Gold Antifade (P36930, Thermo Fisher) and viewed under a Nikon NI-U fluorescent microscope.

### Statistical analysis

Student's *t* test and one way ANOVA with Dunnett's test were used for pairwise comparisons and multigroup comparison respectively. *p* values <0.05 were considered to be significant. All analyses were performed with GraphPad Prism software (Version 6.0). The experiments were not randomized and the investigators were not blinded to allocation during experiments and outcome assessment. Western blot shown is representative of at least two independent experiments.

## Data availability

All the data are included in the article.

## Supporting information

This article contains supporting information.

## Conflict of interest

The authors declare that they have no conflicts of interest with the contents of this article.
