# Supplementary material for: MEKK2 and MEKK3 orchestrate multiple signals to regulate Hippo pathway
Source: J Biol Chem. 2021 Feb 9;296:100400. doi: 10.1016/j.jbc.2021.100400 (PMC7948509; doi:10.1016/j.jbc.2021.100400)
Supplement: Figures S1 to S6 and Tables S1 & S2 [file mmc1.pdf]

Supporting information

MEKK2 and MEKK3 orchestrate multiple signals to regulate Hippo pathway

Jinqiu Lu[1,†], Zonghao Hu[1,†], Yujie Deng[1], Qingzhe Wu[1], Ming Wu[2], Hai Song[1,2*]

Supporting information includes:
Supporting figures S1-S6
Supporting figure legends S1-S6
Supplementary Table 1. qPCR primer, siRNA and guide RNA target sequence.
Supplementary Table 2. Antibody information

Supporting Figure 1

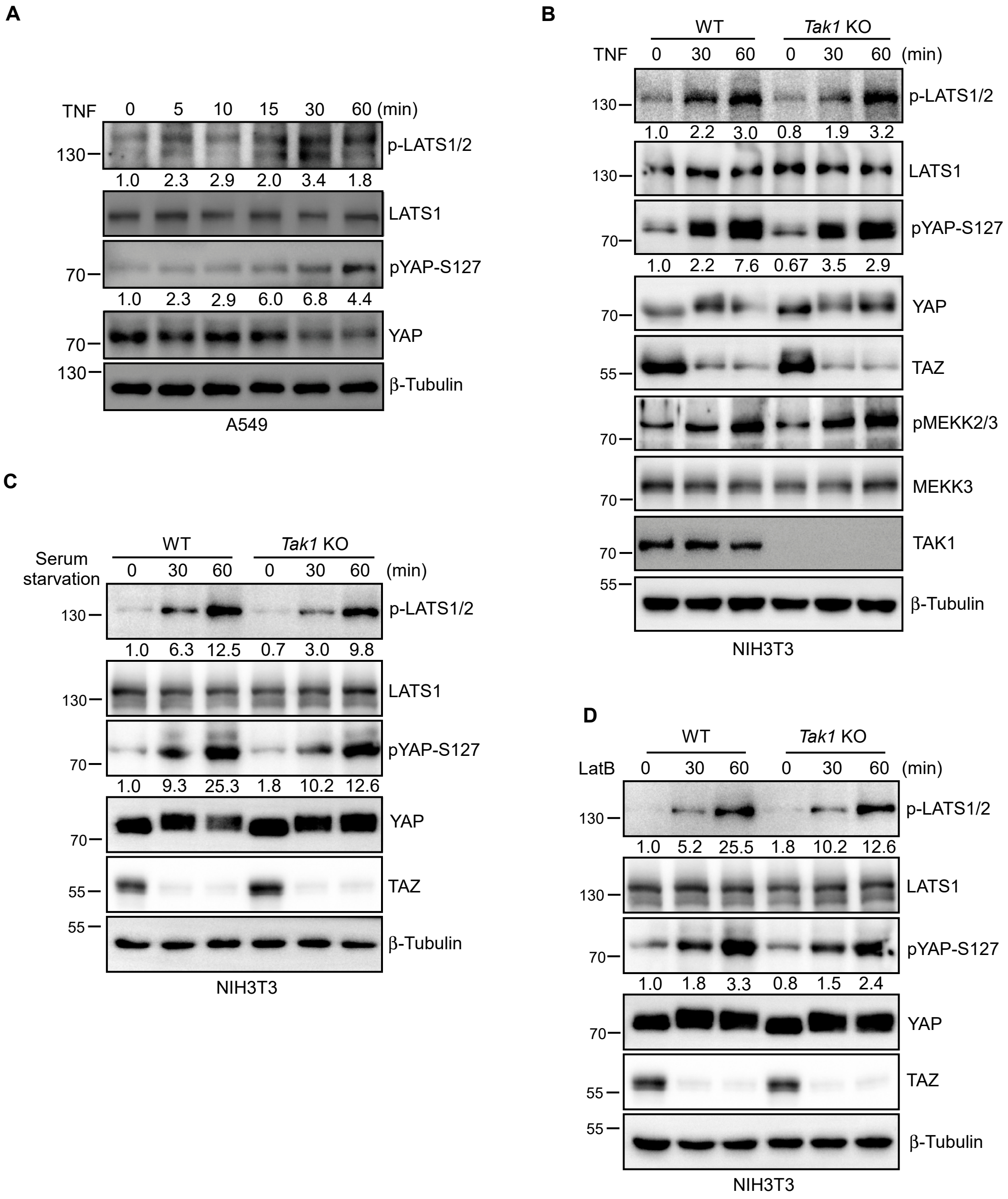

Supporting Figure 2

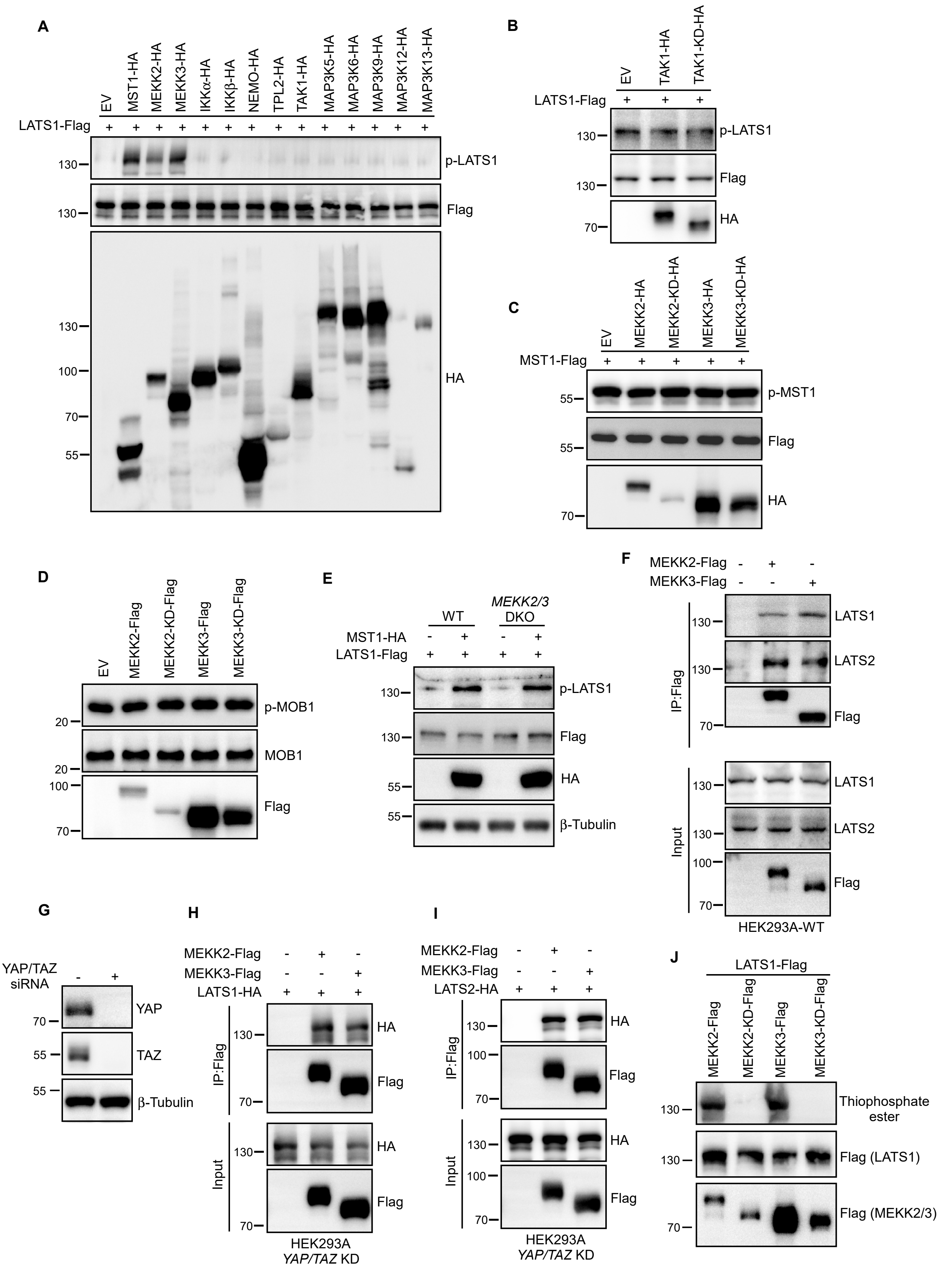

Supporting Figure 3.

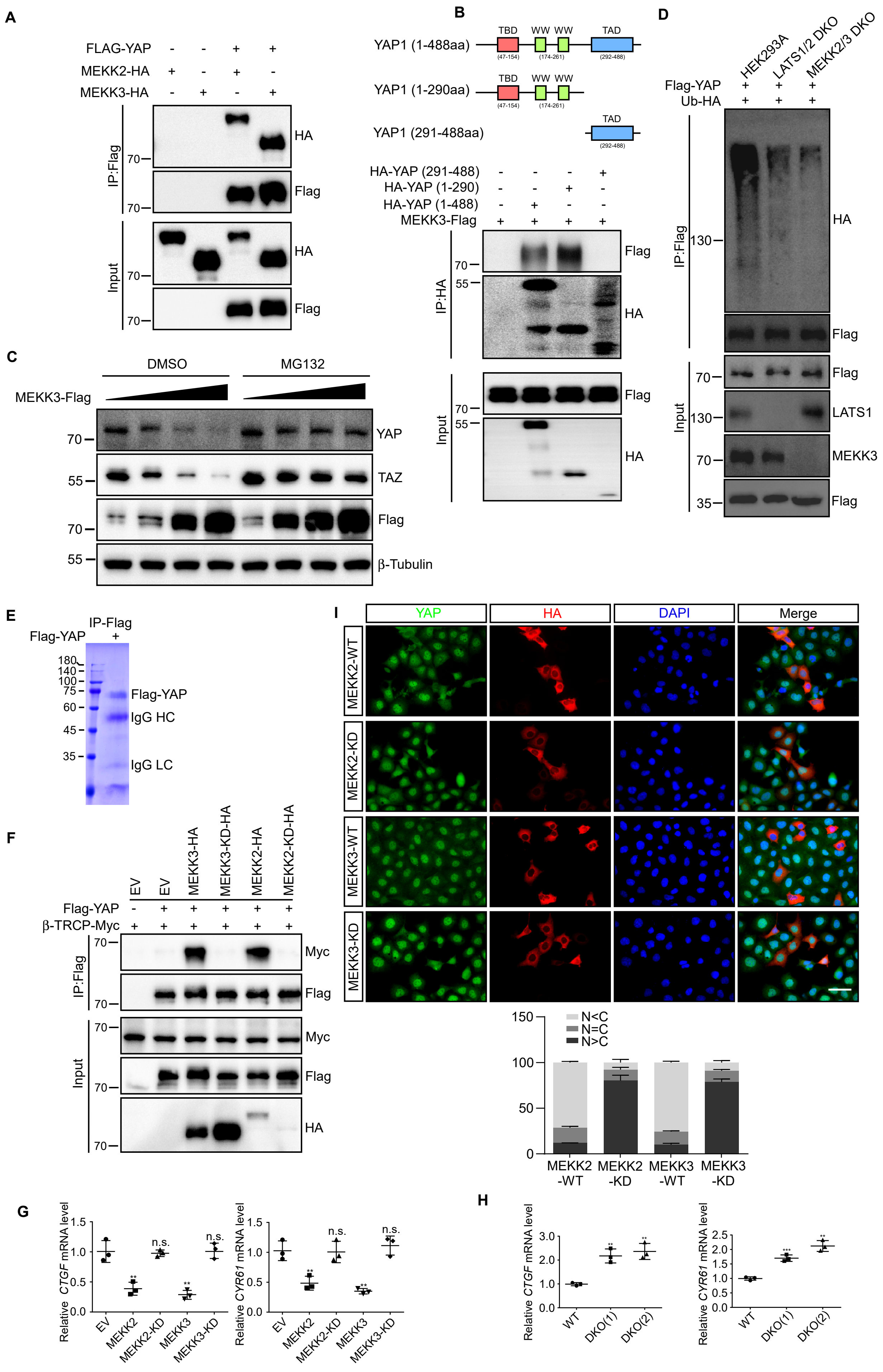

Supporting Figure 4.

A

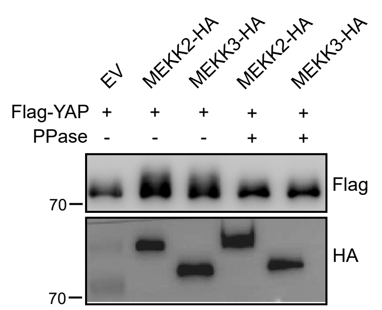

B

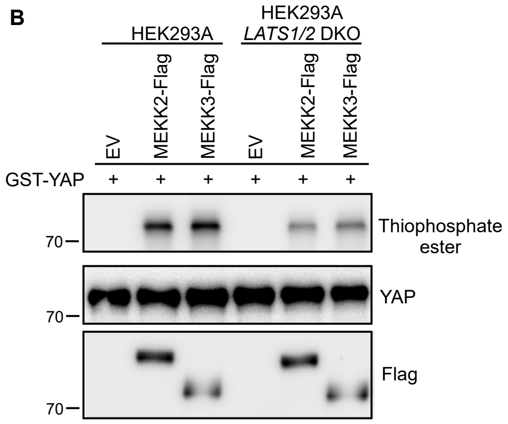

C

Nano LC-MS	YAP5SA+ MEKK3	YAP5SA+ MEKK3-KD
Coverage	65.98%	66.67%
S371	34.69%	4.69%
T412	14.28%	0.00%

D

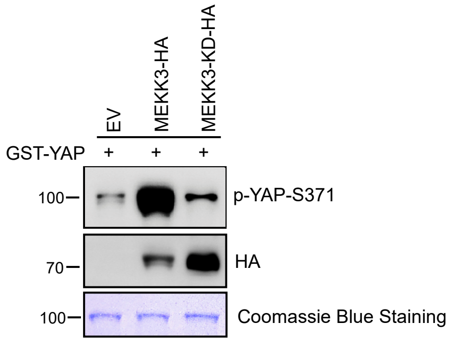

E

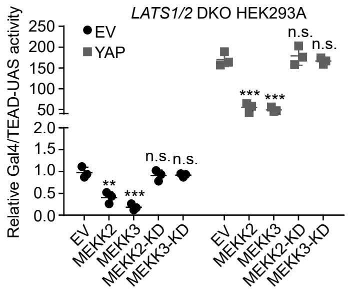

F

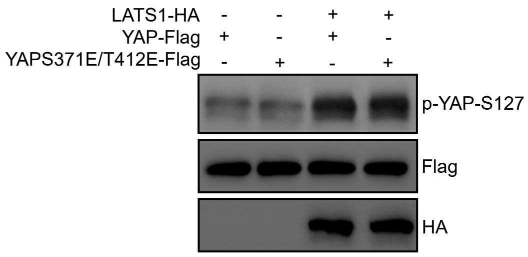

G

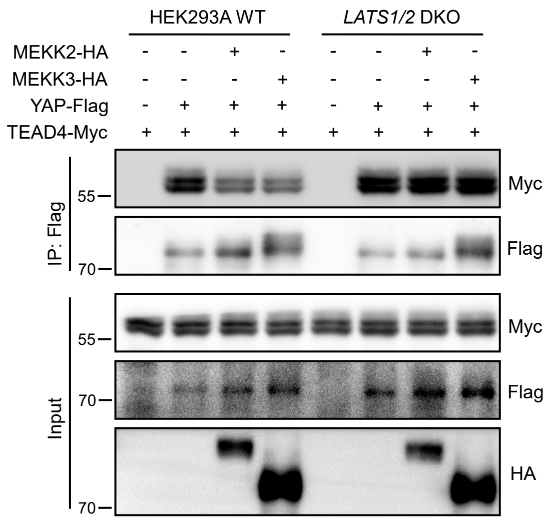

Supporting Figure 5.

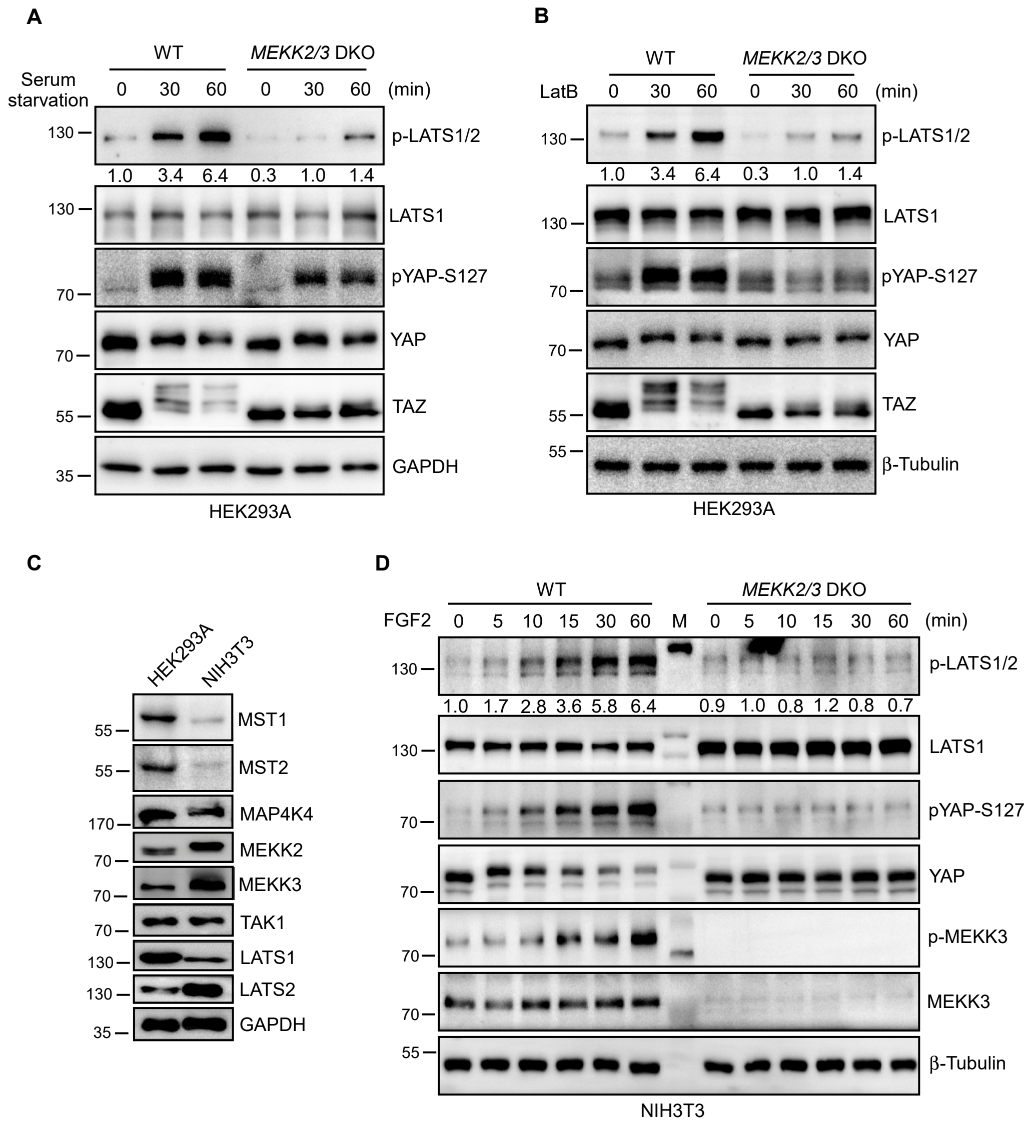

Supporting Figure 6.

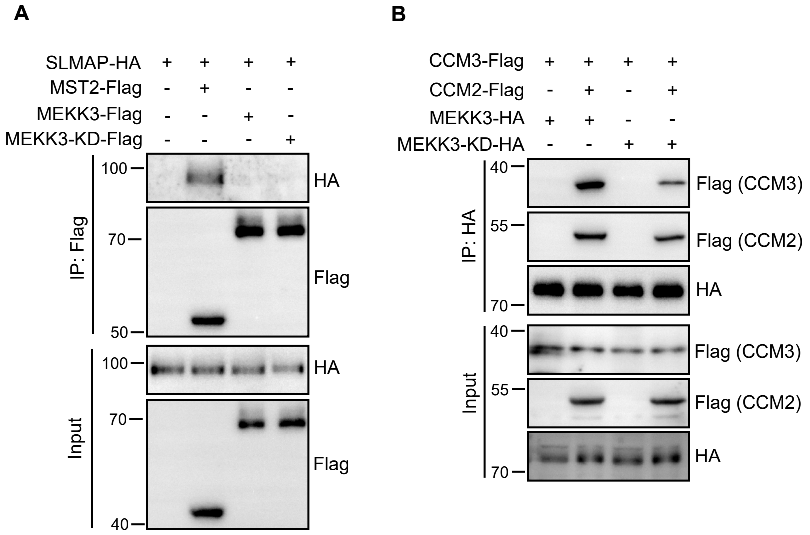

Supporting Figure Legends

Figure S1. **Analysis of LATS activation and YAP phosphorylation in *Tak1* KO NIH3T3 cells in response to different stimuli.**

A. TNF stimulates LATS activation and YAP phosphorylation in *Tak1* KO NIH3T3 cells. WT and *Tak1* KO NIH3T3 cells were cultured in the presence of TNF for the indicated times, and analyzed with indicated antibodies by immunoblotting.
B. Serum starvation induces LATS activation and YAP phosphorylation in *Tak1* KO NIH3T3 cells. WT and *Tak1* KO NIH3T3 cells were serum starved for the indicated times, and analyzed with indicated antibodies by immunoblotting.
C. Actin depolymerization induces LATS activation and YAP phosphorylation in *Tak1* KO NIH3T3 cells. WT and *Tak1* KO NIH3T3 cells were treated with LatB for 30 or 60 mins, and analyzed with indicated antibodies by immunoblotting.
D. TNF stimulates LATS activation and YAP phosphorylation in A549 cells. A549 cells were cultured in the presence of TNF for the indicated times, and analyzed with indicated antibodies by immunoblotting. The western blot was measured using ImageJ to determine the relative intensities of the p-LATS1/2 and pYAP-S127 bands, which were normalized using the LATS1 and YAP proteins respectively. The relative intensities are shown in (A-D).

Figure S2. MEKK2 and MEKK3 associate with LATS1/2 and promote their activation.

A. Characterization of the ability of MAP3K family and NF-κB pathway related kinases to induce LATS1 activation. HEK293A cells were transiently transfected with LATS1-Myc together with vector or MST1-HA, MAP3K family or NF-κB pathway related kinases. LATS1 activation was determined by the phosphorylation status of LATS hydrophobic motif.

B. TAK1 does not promote LATS phosphorylation. HEK293A cells were transiently transfected with LATS1-Flag together with TAK1-HA or TAK1-KD-HA plasmids. LATS1 activation was determined by the phosphorylation status of LATS hydrophobic motif with anti-pLATS antibody.

C. MEKK2/3 do not promote MST1 activation. HEK293T cells were transiently transfected with MST1-Flag together with MEKK2-HA, MEKK2-KD-HA, MEKK3-HA, or MEKK3-KD-HA plasmids. MST1 activation was determined by the phosphorylation status of MST1-Thr183/MST2-Thr180 with anti-pMST1/2 specific antibody.

D. MEKK2/3 do not promote MOB1 phosphorylation. HEK293T cells were transiently transfected with MOB1-HA together with MEKK2-HA, MEKK2-KD-HA, MEKK3-HA, or MEKK3-KD-HA plasmids. MOB1 phosphorylation by MST1 was determined by the phosphorylation status of MOB1-T35 with anti-pMOB1 specific antibody.

E. MST1 phosphorylates LATS1 independent MEKK2/3. Cell lysates from WT and *MEKK2/3* KO HEK293A cells transiently transfected with MST1-HA and LATS1-Flag plasmids as indicated were immunoblotted with indicated antibodies.

F. MEKK2/3 associate with LATS1/2. HEK293T cells were transiently transfected with MEKK2-Flag or MEKK3-Flag plasmids. Immunoprecipitated protein complexes with anti-Flag antibody were subjected to immunoblot with anti-LATS1 and LATS2 antibodies.

G. *YAP/TAZ* knockdown HEK293A cells used in (H) and (I) were analyzed by anti-YAP and TAZ antibodies. HEK293A cells were transfected with siRNA against *YAP* and *TAZ*.

H. LATS1 associates with MEKK2/3 independent of YAP and TAZ. HEK293T cells were transfected with siRNA against *YAP* and *TAZ*, and then transfected with LATS1-HA plasmid together with MEKK2-Flag or MEKK3-Flag plasmids. Immunoprecipitated protein complexes with anti-Flag antibody were subjected to immunoblot with indicated antibodies.

I. LATS2 associates with MEKK2/3 independent of YAP and TAZ. HEK293T cells were transfected with siRNA against *YAP* and *TAZ*, and then transfected with LATS2-HA together with MEKK2-Flag or MEKK3-Flag plasmids. Immunoprecipitated protein complexes with anti-Flag antibody were subjected to immunoblot with indicated antibodies.

J. MEKK2/3 phosphorylate LATS1 in the *in vitro* kinase assay. LATS1-Flag, MEKK2-Flag, MEKK2-KD-Flag, MEKK3-Flag or MEKK3-KD-Flag was transiently expressed in HEK293A cells and immunoprecipitated with anti-Flag antibody individually. The *in vitro* kinase assay was performed using immunoprecipitated LATS1-Flag proteins as substrates in the presence of ATP-γ-S. Total phosphorylation of LATS1-Flag protein was detected by immunoblotting with anti-thiophosphate ester antibody.

Figure S3. MEKK2 and MEKK3 interact with YAP/TAZ and inhibit their function.

A. MEKK2 and MEKK3 interact with YAP independent of LATS1/2. *LATS1/2* DKO HEK293A cells were transiently transfected with Flag-YAP together with MEKK2-HA or MEKK3-HA plasmids. Flag-YAP proteins were immunoprecipitated, and the associated MEKK2-HA or MEKK3-HA proteins were detected by immunoblotting.

B. WW domain of YAP is required for the interaction with MEKK3. A series of YAP deletion mutants were constructed as indicated and transfected into HEK293T cells together with MEKK3-Flag. YAP deletion mutants were immunoprecipitated with anti-HA antibody, and the associated MEKK3-Flag proteins were detected by immunoblotting.

C. MG132 restores endogenous YAP and TAZ protein levels in MEKK3 overexpressing HEK293T cells. HEK293T cells were transiently transfected with increased dose of MEKK3-HA plasmids, and treated with MG132 for 2 h before harvest. YAP and TAZ protein levels were detected by immunoblotting.

D. YAP ubiquitination is reduced in *MEKK2/3* DKO HEK293A cells. Ubiquitination assay of Flag-YAP was performed in WT, *MEKK2/3* DKO and *LATS1/2* DKO HEK293A cells with the overexpression of HA-tagged Ubiquitin treated with MG132 (10 μM) for 2 h before harvest.

E. YAP associated proteins were not immunoprecipitated by anti-Flag antibody using RIPA buffer. Flag-YAP proteins were immunoprecipitated from HEK293A cells lyzed with RIPA buffer, and were stained with Coomassie Blue dye in SDS-PAGE.

F. MEKK2/3 promote the interaction between YAP and β-TRCP. HEK293T cells were transiently transfected with plasmids as indicated. YAP proteins were immunoprecipitated with anti-Flag antibody, and the associated β-TRCP-Myc proteins were detected by immunoblotting.

G. MEKK2/3 inhibit the expression of YAP target genes in HEK293T cells. Empty vector, MEKK2-HA, MEKK2-KD-HA, MEKK3-HA or MEKK3-KD-HA plasmids were transfected into HEK293A cells. Quantification of *CTGF* and *CYR61* mRNA levels normalized with the level of *GAPDH* was performed by quantitative real-time PCR analysis. N=3 independent experiments. **$P < 0.01$.

H. The expression of YAP target genes is increased in *MEKK2/3* DKO HEK293A cells. Quantification of *CTGF* and *CYR61* mRNA levels normalized with the level of *GAPDH* in WT and *MEKK2/3* DKO HEK293A cells was performed by quantitative real-time PCR analysis. N=3 independent experiments. **$P < 0.01$.

I. Overexpression of MEKK2 and MEKK3 induces cytoplasmic translocation of YAP in HEK293A cells. HEK293A cells were transiently transfected with MEKK2-HA, MEKK2-KD-HA, MEKK3-HA or MEKK3-KD-HA plasmids. Localization of YAP, MEKK2 and MEKK3 was determined by immunofluorescence staining with the YAP (green) and HA (red) antibodies. DAPI (blue) was used to visualize cell nuclei. Percentage of YAP cellular localization was shown in the right panel. Scale bars: 20 μm. A total of 100 HA positive cells were analyzed from 3 independent experiments in each panel.

Figure S4. **MEKK2 and MEKK3 phosphorylate YAP and inhibit its transcriptional activity.**

A. MEKK2/3-induced mobility shift of YAP is abrogated after treated with λPPase. Lysates from Flag-YAP, MEKK2-HA or MEKK3-HA expressing HEK293T cells were treated with or without λPPase and immunoblotted with the indicated antibodies.
B. MEKK2/3 phosphorylate YAP in the *in vitro* kinase assay. MEKK2-Flag and MEKK3-Flag proteins purified from WT HEK293A or *LATS1/2* DKO HEK293A cells were incubated with GST-YAP recombinant proteins purified from *E.coli* and subjected to the *in vitro* kinase assay. Phosphorylated YAP was detected with anti-thiophosphate ester antibody.
C. Nano-Liquid Chromatography/Mass Spectrometry analysis of enhanced phosphorylation sites in YAP5SA proteins coexpressed with MEKK3-HA or MEKK3-KD-HA.
D. MEKK3 phosphorylates YAP at S371 in the *in vitro* kinase assay. MEKK3-Flag proteins purified from *LATS1/2* DKO HEK293A cells were incubated with GST-YAP recombinant proteins purified from *E.coli* and subjected to the *in vitro* kinase assay. Phosphorylated YAP was detected with anti-pYAP-S371 antibody.
E. MEKK2/3 inhibit YAP-induced Gal4/TEAD4-luciferase activity in *LATS1/2* DKO HEK293A cells. Luciferase assay of Gal4/TEAD4 reporter activity was assayed in *LATS1/2* DKO HEK293A cells transfected with indicated plasmids together with Gal4/TEAD4 reporter system. N=3 independent experiments. **$P < 0.01$, and ***$P < 0.001$.
F. Phosphorylation of YAP at S371 and T412 does not affect YAP Phosphorylation at S127 by LATS kinases. S371 and T412 of YAP were mutated into glutamic acid. Phosphomimetic of YAP (YAPS371E/T412E) or WT-YAP plasmids were transfected with LATS1-HA into HEK293T cells. Cell lysates were analyzed using anti-p-YAP-S127 antibody.
G. MEKK2/3 reduce the interaction between YAP and TEAD dependent on LATS1/2. HEK293A and *LATS1/2* DKO HEK293A cells were transfected as indicated. YAP was immunoprecipitated by anti-Flag antibody. Associated TEAD4 was detected by Myc antibody.

Figure S5. **MEKK2 and MEKK3 mediate various signals-induced LATS activation.**

A. Serum starvation-induced LATS activation and YAP phosphorylation are compromised in the absence of MEKK2/3 in HEK293A cells. WT and MEKK2/3 DKO HEK293A cells were depleted with serum for 30 or 60 mins, and analyzed with indicated antibodies by immunoblotting.
B. Actin depolymerization-induced LATS activation and YAP phosphorylation are compromised in the absence of MEKK2/3. WT and *MEKK2/3* DKO NIH3T3 cells were treated with LatB for 30 or 60 min, and analyzed with indicated antibodies by immunoblotting.
C. Expression analysis of various kinases in HEK293A and NIH3T3 cells. Total cell lysates of HEK293A and NIH3T3 cells were analyzed with indicated antibodies by immunoblotting.
D. FGF2-induced LATS activation and YAP phosphorylation are inhibited in *MEKK2/3* DKO NIH3T3 cells. WT and *MEKK2/3* DKO NIH3T3 cells were serum-starved overnight and followed by FGF2 stimulation for the indicated time. Total cell lysates were analyzed with indicated antibodies by immunoblotting. The western blot was measured using ImageJ to determine the relative intensities of the p-LATS1/2 bands, which were normalized using the LATS1 proteins respectively. The relative intensities are shown in (S5A, S5B and S5D).

Figure S6. CCM2 and CMM3 form a complex with MEKK3.

A. MEKK3 does not interact with SLMAP. HEK293T cells were transfected with indicated plasmids. MST2-Flag and MEKK3-Flag proteins were immunoprecipitated with anti-Flag antibody, and the associated SLMAP-HA proteins were detected with anti-HA antibody by immunoblotting.

B. MEKK3 forms a complex with CCM2 and CCM3. MEKK3-HA or MEKK3-KD-KA plasmids were co-transfected with CCM2-Flag and CCM3-Flag into HEK293A cells. MEKK3-HA proteins were immunoprecipitated with anti-HA antibody, and the associated CCM2-Flag and CCM3-Flag proteins were detected with anti-Flag antibody.

Supplementary Table 1. qPCR primer, siRNA and guide RNA target sequence.

Name	qPCR primer sequence
h*CTGF*-F	GTTTGGCCCAGACCCAACTA
h*CTGF*-R	CTTCTTCATGACCTCGCCGT
h*CYR61*-F	AGTGGGTCTGTGACGAGGAT
h*CYR61*-R	GGGTTTCTTTCACAAGGCGG
h*GAPDH*-F	TGAAGACGGGCGGAGAGAAA
h*GAPDH*-R	TTCCCGTTCTCAGCCTTGAC
Name	CRISPR targeting sequence:
h*TAK1*-1	GAGTTGTTTGCAAAGCTAAG
h*TAK1*-2	AGAGCCTGATGACTCGTTGT
m*Tak1*-1	CCCGGGTCGGTCCCGCGCCA
m*Tak1*-2	GGGCGCCGCGGGGGATCATG
m*Mekk2*-1	TAAAGATAATGCTGGCCGAC
m*Mekk2*-2	CTGGCCGACTGGCCTTATGA
m*Mekk3*-1	AGCCGACGAACCCGGTTGTC
m*Mekk3*-2	TCCAGATGAGCCGACGAACC
h*MEKK2*-1	CAAGGATAATGCTGGTCGAC
h*MEKK2*-2	CTGGTCGACTGGCCTTATGA
h*MEKK3*-1	TCCGGTGACGTCGGTTCATC
h*MEKK3*-2	TCCAGATGAACCGACGTCAC
Name	siRNA targeting sequence
si-*YAP*-Sense-1	CCACCAAGCUAGAUAAAGAdTdT
si-*YAP*-AntiSense-1	UCUUUAUCUAGCUUGGUGGdTdT
si-*YAP*-Sense-2	GCACCUAUCACUCUCGAGAdTdT
si-*YAP*-AntiSense-2	UCUCGAGAGUGAUAGGUGCdTdT
si-*TAZ*-Sense-1	AGAGGUACUUCCUCAAUCAdTdT
si-*TAZ*-AntiSense-1	UGAUUGAGGAAGUACCUCUdTdT
si-*TAZ*-Sense-2	AGGUACUUCCUCAAUCACAdTdT
si-*TAZ*-Anti-Sense-2	UGUGAUUGAGGAAGUACCUdTdT

Supplementary Table 2. Antibody information

Name	Source	# Catalog	Dilution
Rabbit Anti-Phospho-LATS1/2	CST	8654s	WB (1:1000)
Rabbit Anti-LATS1	CST	3477s	WB (1:1000)<br>IP (1:200)
Rabbit Anti-LATS2	CST	5888S	WB (1:1000)
Rabbit Anti-MOB1	CST	3863S	WB (1:1000)
Rabbit Anti-Phospho-MOB1	CST	8843S	WB (1:1000)
Rabbit Anti-MEKK2	Proteintech	55106-1-AP	WB (1:1000)
Rabbit Anti-MEKK2	Abcam	Ab33918	WB (1:1000)
Rabbit Anti-MEKK3	Proteintech	21072-1-AP	WB (1:1000)<br>IP (1:200)
Rabbit Anti-MEKK3	Abcam	ab40756	WB (1:1000)
Rabbit Anti- Phospho-YAP	CST	4911	WB (1:1000)
Rabbit Anti-YAP	CST	4912s	WB (1:1000)
Mouse Anti-YAP	Santa Cruz	sc-101199	WB (1:1000)<br>IF (1:200)
Rabbit Anti-TAZ	Proteintech	23306-1-AP	WB (1:1000)
Rabbit Anti-NF-κB p65	CST	8242s	WB (1:2000)
Rabbit Anti- Phospho-NF-κB p65	CST	3033s	WB (1:2000)
Rabbit Anti-MAP4K4	Proteintech	55247-1-AP	WB (1:1000)
Rabbit Anti-MST1	CST	3682s	WB (1:1000)
Rabbit Anti-MST2	CST	3952s	WB (1:1000)
Rabbit Anti- Phospho-MST1	CST	3681	WB (1:1000)
Rabbit Anti-RIPK1	CST	3493	WB (1:1000)
Rabbit Anti-TAK1	Abcam	ab109526	WB (1:1000)
Rabbit Anti-CCM2	Proteintech	26270-1-AP	WB (1:1000)
Rabbit Anti-STRN3	Proteintech	23966-1-AP	WB (1:1000)
Mouse Anti-HSP90	Proteintech	60318-1-Ig	WB (1:1000)
Rabbit Anti-β-TUBLIN	Proteintech	66031-1-Ig	WB (1:2000)
Rat Anti-HA	Roche	11867431001	WB (1:1000)
Rabbit Anti-HA	Proteintech	51064-2-AP	WB (1:1000)<br>IF (1:200)
Mouse Anti-Flag	Sigma	F3165	WB (1:2000)
Mouse Anti-MYC	BIOXCELL	BE0238	WB (1:10000)
Rabbit Anti-GAPDH	Proteintech	60004-1-Ig	WB (1:20000)
Rabbit Anti-Thiophosphate ester	Abcam	ab92570	WB (1:1000)
Mouse Anti-GST	Sangon Biotech	D199985	WB (1:1000)
Mouse Anti-Flag magnetic beads	Sigma	M8823	IP (1:200)
Mouse Anti-HA magnetic beads	Thermo Fisher	88837	IP (1:200)
